# Central tolerance shapes the neutralizing B cell repertoire against a persisting virus in its natural host

**DOI:** 10.1073/pnas.2318657121

**Published:** 2024-03-06

**Authors:** Marianna Florova, Tiago Abreu-Mota, Guido C. Paesen, Anna Sophia Beetschen, Karen Cornille, Anna-Friederike Marx, Kerstin Narr, Mehmet Sahin, Mirela Dimitrova, Nivedya Swarnalekha, Jane Beil-Wagner, Natasa Savic, Pawel Pelczar, Thorsten Buch, Carolyn G. King, Thomas A. Bowden, Daniel D. Pinschewer

**Affiliations:** ^a^Division of Experimental Virology, Department of Biomedicine, University of Basel, Basel 4009, Switzerland; ^b^Division of Structural Biology, Wellcome Centre for Human Genetics, University of Oxford, Oxford OX3 7BN, United Kingdom; ^c^Department of Biomedicine, Immune Cell Biology Laboratory, University Hospital Basel, Basel 4031, Switzerland; ^d^Institute of Laboratory Animal Science, University of Zurich, Zurich 8093, Switzerland; ^e^ETH Phenomics Center, ETH Zürich, Zürich 8093, Switzerland; ^f^Center for Transgenic Models, University of Basel, Basel 4001, Switzerland

**Keywords:** persistent viral infection, self-tolerance, viral immune evasion, neutralizing antibodies, B cell receptor editing

## Abstract

Viral mimicry of host cell structures has been postulated to curtail the B cell receptor (BCR) repertoire against persisting viruses through tolerance mechanisms. This concept awaits, however, experimental testing in a setting of natural virus–host relationship. We engineered mouse models expressing a monoclonal BCR specific for the envelope glycoprotein of lymphocytic choriomeningitis virus (LCMV), a naturally persisting mouse pathogen. When the heavy chain of the LCMV-neutralizing antibody KL25 was paired with its unmutated ancestor light chain, most B cells underwent receptor editing, a behavior reminiscent of autoreactive clones. In contrast, monoclonal B cells expressing the same heavy chain in conjunction with the hypermutated KL25 light chain did not undergo receptor editing but exhibited low levels of surface IgM, suggesting that light chain hypermutation had lessened KL25 autoreactivity. Upon viral challenge, these IgM^low^ cells were not anergic but up-regulated IgM, participated in germinal center reactions, produced antiviral antibodies, and underwent immunoglobulin class switch as well as further affinity maturation. These studies on a persisting virus in its natural host species suggest that central tolerance mechanisms prune the protective antiviral B cell repertoire.

## Significance

Tolerance to self represents a functional cornerstone of the vertebrate immune system, enabling the defense against infectious intruders while averting autoimmune disease. Studying the B cell repertoire against a naturally persisting virus of mice we find that precursors of protective, virus-neutralizing B cells are eliminated by receptor editing, a key mechanism of central B cell tolerance. Our findings in a physiological setting of virus–host relationship indicate that persisting viruses subvert protective antiviral B cell responses by mimicking self. These insights can explain the excessively low precursor frequencies of B cells able to neutralize persisting viruses such as HIV, which in return accounts for vastly delayed and inadequate neutralizing antibody responses to vaccination against or infection by these viruses.

Neutralizing antibodies (nAbs) represent a cornerstone of antiviral host defense and vaccine protection. Accordingly, delayed and inadequate nAb responses to chronic viral diseases facilitate pathogen persistence and can thwart antibody-based vaccination efforts (1–6). Prototypic examples are HIV and hepatitis C virus (HCV) in humans and lymphocytic choriomeningitis virus (LCMV) in mice (1, 7).

Unlike for acute viral infections such as SARS-CoV-2, influenza A virus, or vesicular stomatitis virus (8–11), it appears that the immune system cannot rely on germline antibody sequences to neutralize the envelope proteins of HIV or LCMV. It rather depends on the hypermutation-based refinement of a low-affinity repertoire. Germline precursors of HIV broadly nAbs (bnAbs) exhibit poor to undetectable binding to the native HIV envelope protein trimer (12–14). Similarly, LCMV nAbs—such as the archetypal antibody KL25 analyzed here—when reverted to their putative unmutated ancestor (UA) sequence are unable to neutralize LCMV and bind to its glycoprotein (GP) with ~100-fold lower affinity than their final hypermutated progeny (15). Accordingly, HIV bnAbs in humans and LCMV nAbs in mice are preferentially elicited under conditions of long-term unchecked viremia (15–17), i.e., when high amounts of persisting viral antigen fuel the continuous evolution of B cells in germinal centers (GCs). The recruitment of low-affinity B cells into the GC response can, however, be inefficient, notably when precursor frequencies of such cells are low (18). The mechanisms accountable for the apparent lack of a germline antibody repertoire able to neutralize persisting viruses have mostly remained obscure but promise important clues to understand the delay and inadequacy of nAb responses elicited by these infectious intruders.

A significant proportion of the B cell receptor (BCR) repertoire, which is randomly generated in the bone marrow, exhibits autoreactivity and can result in the respective cells’ deletion at the immature B cell stage (19, 20). The main mechanism warranting self-tolerance of the mature B cell compartment consists, however, in receptor editing by autoreactive immature B cells during development (21–23). BCR ligation by self-antigen on immature B cells in the bone marrow can lead to surface IgM downregulation and developmental arrest, precluding the cells’ bone marrow egress and their subsequent maturation into IgD^hi^ recirculating cells (24, 25). Consequent cessation of tonic surface IgM signaling causes the cells’ “back-differentiation” toward a pre-B cell stage and re-expression of recombination-activating genes (RAG1 and RAG2), culminating in the resumption of light chain rearrangement (26–28). Thereby, immature B cells that are autoreactive remain IgM^lo^IgD^neg^ and undergo further BCR rearrangement until a nonautoreactive BCR is expressed. Alternatively, the autoreactive receptor can be diluted by the expression of additional chains (29–31). In conjunction with microbial mimicry of self, such tolerance mechanisms have been proposed to account for the sparseness of certain antimicrobial specificities in the pre-immune B cell repertoire (32–34). Accordingly, HIV bnAbs can be polyreactive and/or autoreactive (20, 35, 36), and the introduction of V(D)J elements from several HIV-bnAbs or from these antibodies’ UA, respectively, into the mouse germline resulted in widespread B cell deletion and/or significant receptor editing (18, 20, 36–40). Certain bnAb-expressing B cells that escaped central tolerance mechanisms down-regulated surface IgM (36), a classical feature of autoreactive B cells (41, 42), which is the result of an altered transcriptional program (43–46). Functional inactivation or hyporeactivity, collectively termed anergy (41, 47, 48), has also been observed in HIV bnAb-expressing B cells (37). Finally, autoimmune mouse strains were found to mount HIV-nAb responses either spontaneously or upon nonspecific immune stimulation (49–51), altogether suggesting that HIV may evade the immune system by mimicking self, thus provoking clonal elimination of virus-reactive B cells (20). The HIV bnAb b12 represents a notable exception to this pattern. It displayed autoreactivity in vitro (35) but failed to trigger significant receptor editing or deletion in developing B cells (52). Species-specific differences in autoantigen sequence and/or abundance (53) may thus confound the investigation of B cell development, fate, and reactivity when HIV-bnAbs of human origin are studied in BCR-engineered mice.

**Fig. 1.**
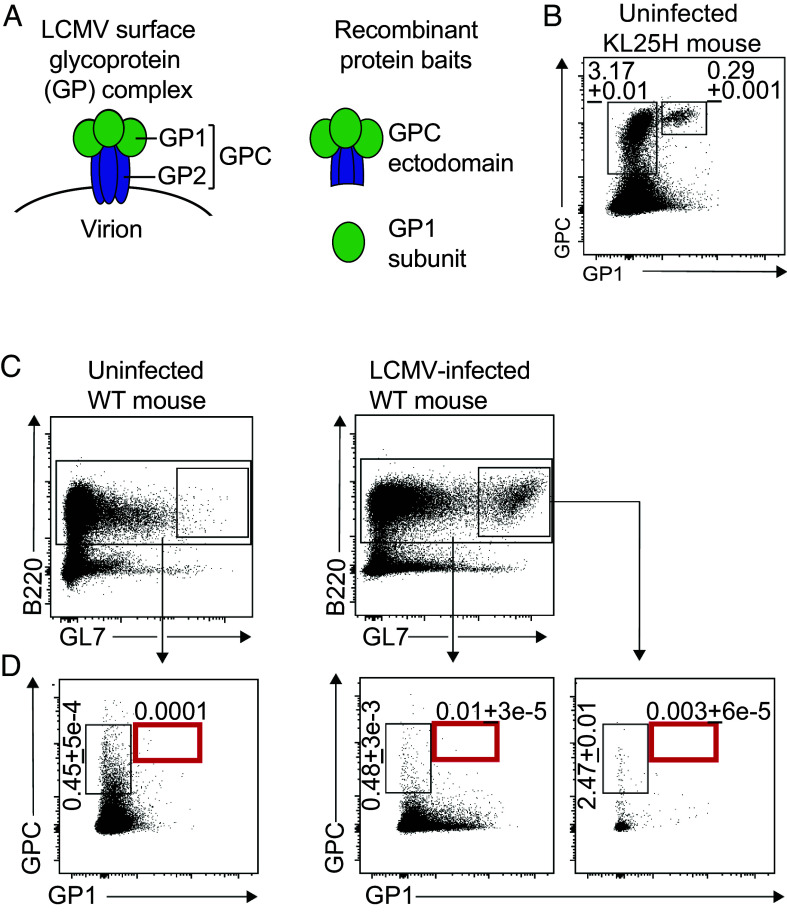
Very low frequencies of LCMV GP1-binding B cells in wild-type mice. (*A*) Schematic of the membrane-anchored LCMV surface GP complex. Heterotrimers consisting of the outer globular GP1 domain, noncovalently associated with the transmembrane GP2 stalk and the stable signal peptide (not displayed), form trimeric complexes on infectious virions. As derivatives thereof, a recombinantly expressed, noncleavable extracellular domain of the GPC ectodomain and a recombinantly expressed GP1 domain, each of them labeled with different fluorophores, were generated as baits for flow cytometry (FACS) use. (*B*) B220^+^ splenocytes of KL25H mice were analyzed for GPC and GP1 binding (see *SI Appendix*, Fig. S1*A* for gating). Numbers in the exemplary FACS plot indicate percentages of gated cells. Number of biological replicates per experimental group (n) = 6. Representative results from one out of two experiments. (*C* and *D*) WT mice were infected with rCl13/WE, uninfected mice served as negative controls. 28 d after infection the total splenic B220^+^ B cell population (see *SI Appendix*, Fig. S1*B* for gating) of infected mice and of uninfected controls (*C*) was analyzed by FACS for GPC and GP1 binding (*D*). A GL7^+^ GC B cell subset was detected in infected but not in uninfected mice. Numbers in exemplary FACS plots indicate the percentage of gated cells as mean ± SEM of 3 to 5 mice (n). One representative of two experiments (N) is shown.

LCMV infection in mice represents a prototypic and physiological example of virus–host relationship in persistent viremic infection. With hallmarks such as T cell exhaustion, it is widely studied as a model of immune subversion in persistent infection (54–56). The LCMV envelope GP is synthesized as a GPC precursor protein, which is posttranslationally cleaved into the protein’s stable signal peptide, an outer globular GP1 domain, and a transmembrane stalk denominated GP2, all of which remain associated in a heterotrimeric GP complex decorating infectious virions (Fig. 1*A*). In analogy to the HIV and HCV envelope proteins, GP1 as a main target of LCMV-nAbs carries a dense glycan shield for nAb evasion (57–62). Accordingly, high-affinity binding by prototypic LCMV-nAbs such as KL25 relies on interactions with defined amino acid residues as well as with specific glycans (59, 63).

Here, we investigated the development, phenotype, and antigen responsiveness of murine B cells expressing either the prototypic mouse-derived LCMV-nAb KL25 or a variant thereof, wherein the light chain was reverted to its UA sequence. We found that B cells with GP1-binding capacity exhibited features commonly associated with autoreactivity such as receptor editing and IgM receptor downregulation. Thereby, our data suggest that protective LCMV-nAb responses arise at least in part from a centrally pruned, autoreactive repertoire.

## Results

### Very Low Frequencies of LCMV GP1-Binding B Cells in Wild-Type Mice.

With the aim of detecting LCMV GP1-binding B cells by flow cytometry, we generated two recombinant protein baits, both based on the LCMV WE strain GP (WE-GP), which we labeled with different fluorophores (Fig. 1*A*). One bait consisted of an isolated GP1 domain, which was combined with a noncleavable version of the GPC ectodomain in an attempt to reduce nonspecific background signal. To test and validate these tools, we used oligoclonal KL25H mice carrying an Ig heavy chain VDJ knock-in derived from the archetypal GP1-binding, LCMV-neutralizing antibody KL25 (15, 64–68) (see *SI Appendix*, Table S1 for a synopsis of immunoglobulin-engineered mouse lines studied in this manuscript). Random pairing of the knock-in KL25 heavy chain with endogenous light chains yields a diverse BCR repertoire yet ascertains elevated frequencies of GP1-binding B cells (63, 65, 69). Approximately 4 to 5% of splenic KL25H B cells bound GPC (Fig. 1*B* and *SI Appendix*, Fig. S1*A*). A minor subset thereof, ~0.3% of the total B cell population, bound GP1 in addition to GPC, thus likely representing bona fide GP1-binding cells. In contrast, the frequency of GPC^+^GP1^–^ single-binding cells of naive WT mice was in the range of 0.5% of B220^+^ B cells while GPC+GP1+ double-binding B cells were practically undetectable (Fig. 1 *C* and *D* and *SI Appendix*, Fig. S1*B*). Next, we infected WT mice with an engineered LCMV clone 13 that expresses the WE-GP [rCl13/WE (59)] and analyzed their spleens 28 d later. GPC-binding cells were enriched to ~2.5% among GL7+ GC B cells (Fig. 1 *C* and *D*), but GPC^+^GP1^+^ double-binding cells remained below detection limit, still, and neither population was detectably enriched in the total B220^+^ B cell compartment. Altogether, these findings suggested that GP1-binding B cells are very rare, both in naive and LCMV-immune mice.

### GP1-Binding KL25H B Cells Protect against LCMV.

**Fig. 2.**
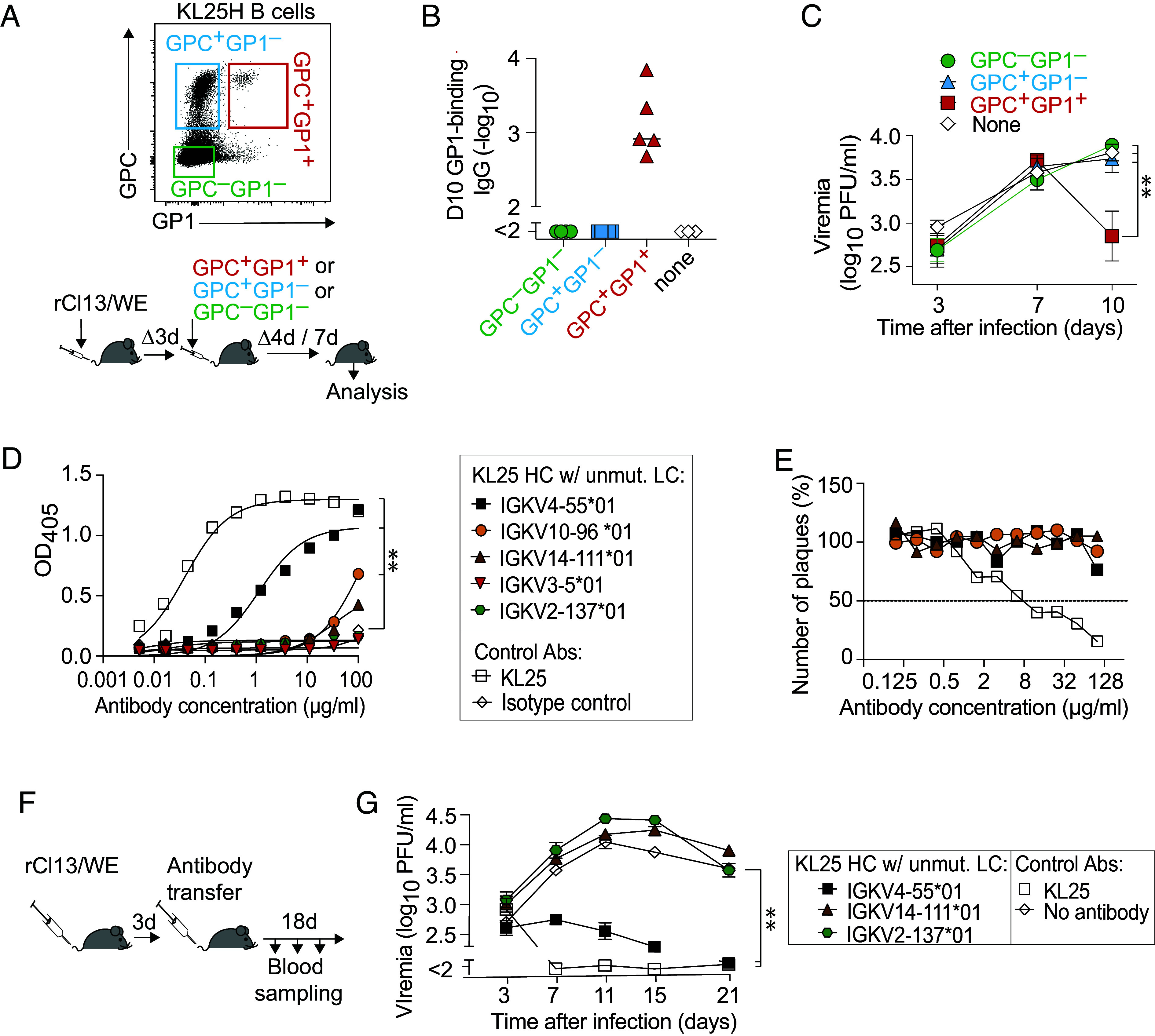
GP1-binding KL25H B cells protect against LCMV. (*A*–*C*) Representative FACS plot of GPC and GP1 binding by KL25H cells (*A*) and schematic of the experimental design for (*B* and *C*); Recipients were infected with rCL13/WE. Three days later GPC+GP1+, GPC+GP1–, and GPC–GP1– B cells (populations defined as shown in *A* and in *SI Appendix*, Fig. S2*A*) were sorted from KL25H mice, and 10,000 of each type were adoptively transferred. Serum antibodies were measured on d10 (*B*) and viremia was determined at the indicated time points (*C*). Symbols in *B* represent individual mice; in *C*, the mean ± SEM of n = 4 to 5 mice is shown. N = 2. One-way ANOVA with Dunnett’s posttest for multiple comparisons in *C*. **P < 0.01. (*D* and *E*) GP1 binding (*D*) and rCl13/WE neutralization (*E*) by recombinantly expressed antibodies consisting of the KL25 heavy chain and the identified light chains. Symbols represent the mean of 2 technical replicates. N = 2. Two-way ANOVA with Dunnett’s posttest for multiple comparisons was performed in *D*, and antibodies exhibiting significantly higher binding than isotype control are indicated. **P < 0.01. (*F*) Schematic of the experimental design for *G*. WT mice were infected with rCl13/WE on day 0, followed by passive immunization with the indicated antibodies on day 3 as described previously (67). (*G*) Viremia was monitored. Symbols show means ± SEM of n = 4 to 5 mice per group. N = 2. Two-way ANOVA with Dunnett’s posttest for multiple comparisons was performed for d7 to d21 values. Treatment groups exhibiting significantly lower viral loads than isotype control-treated mice are indicated. **P < 0.01.

KL25H B cells have been shown to suppress viremia after LCMV infection (63, 70). To test how GPC and GP1 binding relates to the protective antiviral capacity of KL25H B cells, we sorted GPC^+^GP1^+^, GPC^+^GP1^–^, and GPC^–^GP1^–^ B cell populations of KL25H mice for adoptive transfer (Fig. 2*A* and *SI Appendix*, Fig. S2*A*). As recipients we used animals that had been infected 3 d prior with rCl13/WE (Fig. 2*A*). Seven days after cell transfer (d10 of the experiment), the recipients of GPC+GP1+ KL25H B cells exhibited GP1-binding antibody titers, as expected, whereas such antibodies were undetectable in all other groups, confirming that GPC^+^GP1^+^ staining by flow cytometry identified GP1-binding B cells (Fig. 2*B*). More importantly even, the transfer of GPC^+^GP1^+^ KL25H B cells suppressed viremia, whereas viral loads in blood of mice receiving GPC^+^GP1^–^ or GPC^–^GP1^–^ B cells were not different from controls without adoptive cell transfer (Fig. 2*C*).

These findings prompted us to try and identify exemplary light chains expressed by GP1^+^GPC^+^ KL25H B cells. By RT-PCR cloning from a pool of FACS-sorted GP1^+^GPC^+^ B cells (compare Fig. 2*A* and *SI Appendix*, Fig. S2*A*), we recovered five different Ig kappa VJ elements (*SI Appendix*, Fig. S2*B*). Among them was also an Ig-kappa V4-55*01/J4 sequence, which corresponded to the UA of the KL25 light chain. Each one of the five different Ig kappa VJ elements was recombinantly expressed in conjunction with the KL25 heavy chain to create antibodies that mimicked the receptors on GP1^+^GPC^+^ KL25H B cells. Two of these five light chain combinations yielded antibodies that bound to GP1 at levels that were significantly above isotype control (Fig. 2*D*), spanning more than two orders of magnitude in binding capacity. The KL25 heavy chain in conjunction with its UA light chain (Ig-kappa V4-55*01/J4; subsequently also referred to as KL25_UA_) bound best but still ~100-fold less than the fully hypermutated KL25. Accordingly, neither of the antibodies containing one of the cloned light chains reached 50% rCl13/WE neutralizing activity when tested at concentrations of up to 128 µg/mL (Fig. 2*E*). We performed passive immunization on day 3 after rCl13/WE infection to test the antiviral efficacy of three of these antibodies (Fig. 2 *F* and *G*). The WT KL25 reference antibody suppressed viremia to below detection limits within 4 d after administration (d7 of the experiment). Significant suppression of viral loads, albeit less immediate, was also observed in mice receiving KL25_UA_, whereas the two other antibodies, which had failed to show significant GP1 binding, did not suppress viremia to below the levels of untreated control mice (Fig. 2*G*, compare Fig. 2 *D* and *E*). Taken together, these results indicated that the rare population of GPC^+^GP1^+^ B cells in KL25H mice represented the most antivirally protective subset. Moreover, the KL25_UA_ specificity seemed well represented among GPC^+^GP1^+^ B cells of KL25H mice and suppressed viral loads when passively administered.

### A Substantial Proportion of KL25_UA_-Expressing B Cells undergo Receptor Editing.

**Fig. 3.**
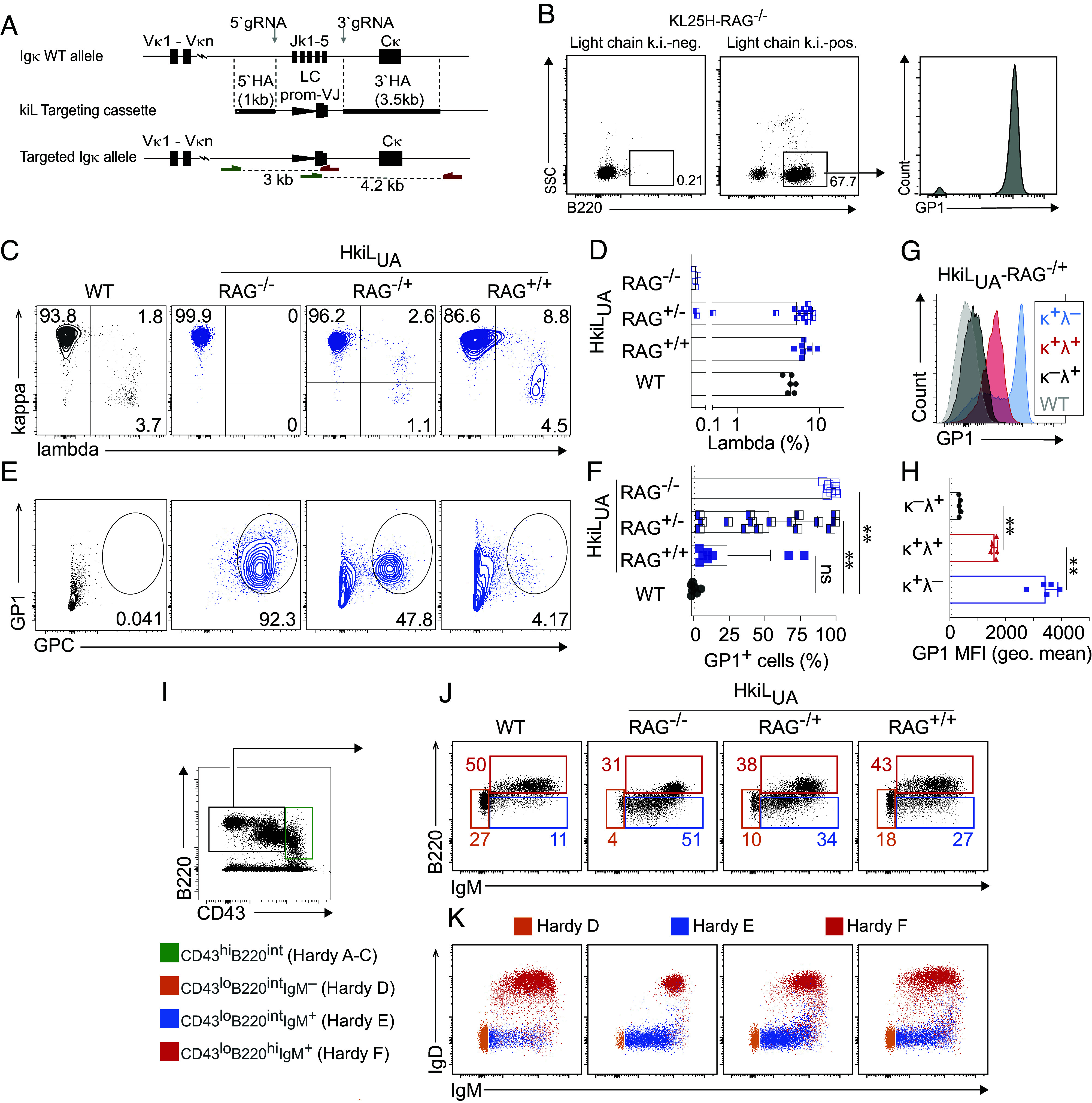
A substantial proportion of KL25_UA_-expressing B cells undergo receptor editing. (*A*) Targeting strategy for the insertion of the KL25_UA_ and KL25 VκJκ exons, respectively, into the mouse immunoglobulin kappa locus. The vector comprises 5′ and 3′ homology arms (5′HA and 3′HA), a kappa light chain promoter (triangle) followed by the VκJκ exons and splice donor site of the KL25 antibody. The PCR genotyping strategy yielding 3 kb and 4.2 kb amplicons (*SI Appendix*, Fig. S3*A*) is depicted. (*B*) Representative FACS plots from blood of founder animals after intercrossing to a RAG2^−/−^ background. Numbers in FACS plots indicate the percentage of gated cells. GP1-binding by B220^+^ B cells of the light chain k.i.-positive founder is shown in the histogram plot. (*C* and *D*) Representative FACS plots (*C*) of Ig kappa and lambda expression on mature splenic B cells (*SI Appendix*, Fig. S3*B*). Symbols in *D* report the percentage of lambda^+^ B cells in individual mice; bars show means ± SEM. (*E* and *F*) Representative FACS plots (*E*) and percentage of GPC+GP1+ splenic mature B cells (see *SI Appendix*, Fig. S3*B* for gating). Symbols in *F* represent individual mice; bars show means ± SEM. (*G* and *H*) GP1 binding by HkiL_UA_-RAG^+/–^ B cells, either κ^+^λ^–^, κ^+^λ^+^, or κ^–^λ^+^. GP1 nonbinding polyclonal B cells from WT mice (gray shaded) are shown as negative control. A FACS histogram from one representative mouse is shown in *G*; symbols in *H* represent individual mice with bars showing the mean + SEM. (*I*) The bone marrow was pre-gated as shown in *SI Appendix*, Fig. S3*C*, and CD43^hi^B220^int^ cells (Hardy *A*–*C*; green gate) were differentiated from the CD43^lo^B220^int/hi^ compartments (Hardy *E* and *F*) to be further analyzed in *J* and *K*). (*J*) B cell developmental stages Hardy *D*–*F* were differentiated as indicated in *I*. Representative FACS plots are shown. See *SI Appendix*, Fig. S3*C* for gating. (*K*) Color-coded superposition of Hardy *D*–*F* developmental stages as gated in *I* for determination of stage-dependent surface IgM and IgD expression. Symbols in *D* and *F* report individual mice analyzed in three independent experiments. One-way ANOVA with Dunnett’s posttest for multiple comparisons in *F* and *H*. **P < 0.01.

These findings prompted us to interrogate the behavior of B cells expressing the KL25_UA_ antibody as their BCR. We used CRISPR/Cas9-based genome targeting to integrate the KL25_UA_ light chain VJ element into the kappa light chain locus (Fig. 3*A* and *SI Appendix*, Fig. S3*A*). The targeting was performed in recombination-activating gene 2 heterozygous (RAG2^+/–^) KL25H oocytes such that after backcrossing to a RAG2^−/−^ background, correctly targeted offspring were readily identified by the animals’ B220^+^ B cell population that was absent in littermates lacking the light chain (Fig. 3*B*). As expected, virtually all B cells of the resulting RAG2-deficient HkiL_UA_ mice (HkiL_UA_-RAG^−/−^) expressed a kappa light chain and bound GP1 (Fig. 3 *C*–*F* and *SI Appendix*, Fig. S3*B*). RAG-dependent receptor editing represents, however, a main mechanism of repertoire selection in B cell ontogeny, and even partial RAG deficiency or haploinsufficiency in RAG hemizygotes can facilitate the maturation and bone marrow egress of autoreactive B cells by reducing the rate of receptor editing (71–74). To investigate to which extent the HkiL_UA_ BCR was subject to receptor editing, we bred HkiL_UA_ knock-in mice to a RAG2-heterozygous or to a RAG2-wild-type background with either one or two functional RAG2 alleles, respectively (HkiL_UA_-RAG^+/−^ and HkiL_UA_-RAG^+/+^). In these animals, lambda chain expression was detected in up to ~10% of B cells (Fig. 3 *C* and *D*). Accordingly, the proportion of B cells binding GP1 varied substantially between individual HkiL_UA_-RAG^+/−^ animals, spanning a range from less than ten to almost one hundred percent (Fig. 3 *E* and *F*), and most HkiL_UA_-RAG^+/+^ mice had less than twenty percent GP1^+^ B cells. κ^+^λ^+^ double-expressing HkiL_UA_-RAG^+/−^ B cells bound less GP1 than the majority of B cells expressing only κ (Fig. 3 *G* and *H*), suggesting that the coexpressed λ chain diluted the knocked-in κ chain (29–31). Moreover, the population of κ single-positive B cells exhibited inhomogeneous GP1 binding. Besides a peak of GP1^hi^ cells, also a subset of κ^+^ λ^–^ single-positive HkiL_UA_-RAG^+/−^ B cells bound intermediate levels of GP1, similar to κ^+^ λ^+^ double-positive cells, suggesting coexpression of a second, unrelated kappa chain from the nontargeted wild-type allele. Altogether, these findings indicated that the KL25_UA_ BCR was prone to receptor editing, suggesting autoreactivity and prompting us to analyze the B cell development in the bone marrow of HkiL_UA_ mice. Pro-B cells of mice with a germline-encoded, pre-rearranged BCR often skip the pre-B cell developmental stage (75, 76) and transition virtually directly to an immature CD43^lo^B220^int^IgM^hi^IgD^–^ [Hardy E (77)] phenotype. When expressing an autoreactive receptor the cells are, however, retained at the immature stage, down-regulate IgM, and subsequently can undergo deletion unless rescued by receptor editing (21, 23, 78). The bone marrow of HkiL_UA_ mice exhibited a substantial immature B cell compartment suggesting retention at this stage. Moreover, immature HkiL_UA_ B cells covered the full spectrum of IgM levels seen in WT controls (Fig. 3 *I*–*K* and *SI Appendix*, Fig. S3*C*), altogether supporting the concept that HkiL_ua_ B cells exhibited autoreactivity and underwent receptor editing.

### Insignificant Receptor Editing But Low Surface IgM in B Cells Expressing a Hypermutated KL25 BCR.

**Fig. 4.**
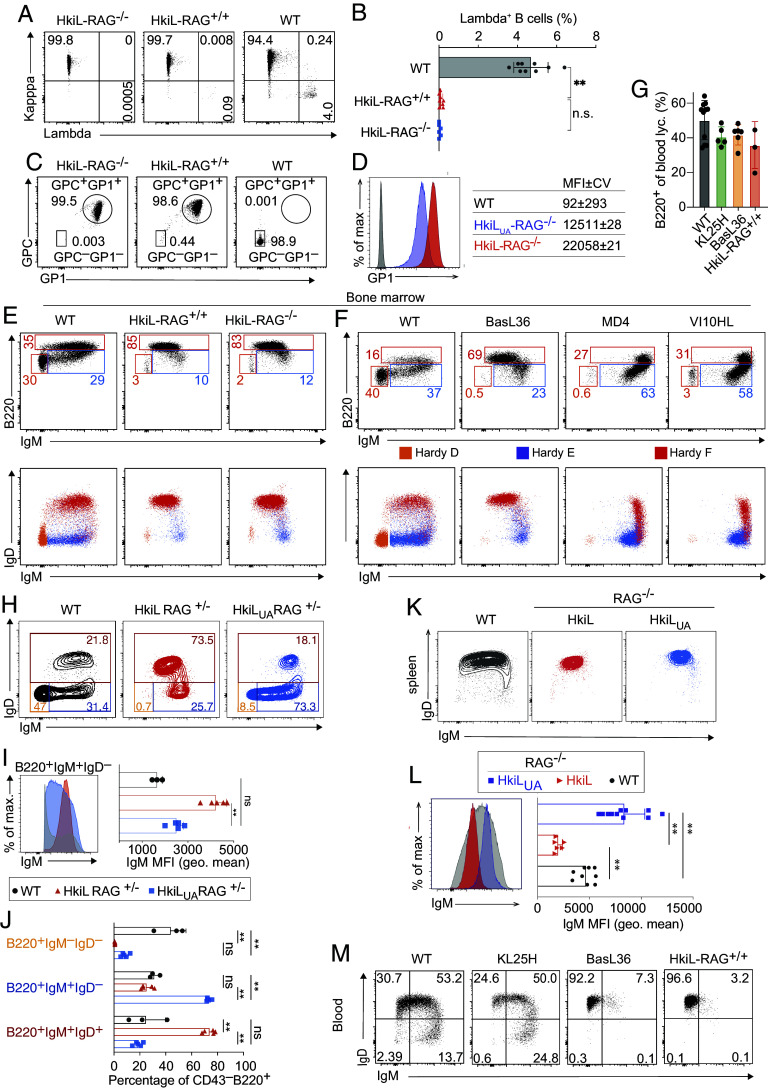
Insignificant receptor editing but low surface IgM on B cells expressing a hypermutated KL25 BCR. (*A* and *B*) Representative FACS plots (*A*) of Ig kappa and lambda expression on mature splenic B cells (see *SI Appendix*, Fig. S3*B* for gating). Symbols in *B* report the percentage of lambda^+^ B cells in individual mice; bars show means ± SD. (*C*) Representative FACS plots showing the percentage of GPC^+^GP1^+^ B cells (see *SI Appendix*, Fig. S3*B* for gating) in the spleen. (*D*) GP1 binding by peripheral blood B cells from the indicated types of mice under conditions of limiting GP1 staining concentrations. MFI is reported as mean ± coefficient of variation. Representative histogram plots are shown. Data from one representative out of three similar experiments are displayed. (*E* and *F*) The *Upper* row shows exemplary FACS plot pre-gated on B220^+^CD43^lo^ bone marrow cells (compare Fig. 3*I* with pre-gating shown in *SI Appendix*, Fig. S3*C*) with gates for pre-B cells (orange), immature B cells (blue), and mature B cells (red). The *Bottom* row shows a color-coded superposition of pre-B, immature B, and mature B cells as gated in the respective panels above for determination of stage-dependent surface IgM and IgD expression. (*E* and *F*) correspond to separate experiments. n = 3 to 4, N = 2. See *SI Appendix*, Fig. S4 *A* and *B* for further information on BasL36 and VI10HL mice. Numbers indicate the percentage of gated cells. (*G*) Percentage of B cells among peripheral blood lymphocytes of KL25 BCR transgenic and/or knock-in mice and WT controls. Symbols represent combined data from individual mice collected in two separate experiments with mean ± SEM. (*H*–*J*) Concatenated FACS plots from the bone marrow of 3 to 5 mice in each group are shown in (*H* and *I*; see *SI Appendix*, Fig. S4*E* for gating). Symbols in *I* and *J* represent individual mice; bars show the mean ± SD. N = 2. (*K* and *L*) Representative FACS plots of surface IgM and IgD expression on B cells in peripheral blood (*K*, see *SI Appendix*, Fig. S3*B* for gating strategy) and of IgM expression and IgM MFI (*L*). Symbols in bar charts to *L* represent individual mice, showing combined data from three independent experiments. (*M*) Surface IgM and IgD expression of peripheral blood B cells (see *SI Appendix*, Fig. S4 *C* and *I* for gating and quantification) of the indicated KL25 BCR-expressing mouse models. One representative FACS plot from a total of 8 to 10 mice analyzed in three independent experiments is shown. See *SI Appendix*, Fig. S4*D* for an analogous analysis from the spleen. Numbers in the FACS plots to (*A*, *C*, *E*, *F*, *H*, and *M*) indicate the percentage of gated cells. One-way ANOVA with Dunnett’s posttest and Tukey’s post hoc analysis was used for multiple comparisons in *I* and *L*, respectively; two-way ANOVA with Tukey’s posttest in *J*. **P < 0.01.

Wide-spread receptor editing and a low percentage of GP1-binding B cells in HkiL_UA_-RAG^+/+^ mice contrasted with earlier observations of a high proportion of GP1-binding B cells in mice carrying the KL25H knock-in in conjunction with a transgenic KL25 light chain (79). To explore the mechanisms underlying this discrepancy, we used Crispr/Cas9 technology to knock-in the hypermutated KL25 light chain VJ element into the autologous kappa chain locus of KL25H-RAG^−/−^ oocytes (compare Fig. 3*A*). Virtually all B cells of the resulting HkiL-RAG^−/−^ mice expressed the kappa light chain and bound GP1, as expected (Fig. 4 *A* and *C*). Higher GP1 binding affinity of HkiL-RAG^−/−^ as compared to HkiL_UA_-RAG^−/−^ B cells was evident in a higher GP1 staining intensity when conducted under conditions of limiting GP1 concentrations (Fig. 4*D*). An additional and even more striking difference between the two receptors was, however, noted when the HkiL BCR was bred to a RAG-sufficient background (HkiL-RAG^+/+^). Unlike in HkiL_UA_-RAG^+/+^ B cells (compare Fig. 3*C*), lambda chain expression was virtually undetectable on HkiL-RAG^+/+^ B cells while ~4% of WT control B cells were lambda^+^ (Fig. 4 *A* and *B*). Moreover, ~99% of HkiL-RAG^+/+^ B cells bound GP1 (Fig. 4*C*), altogether indicating that B cells expressing the hypermutated KL25 receptor were virtually exempt from receptor editing. Accordingly, Hardy fraction E immature B cells in the bone marrow of both RAG-deficient and -sufficient HkiL B cells expressed uniformly high levels of surface IgM (Fig. 4*E*). In addition to the HkiL light chain knock-in model, which by genetic design is amenable to light chain editing, we used KL25H mice as a basis for a KL25 light chain transgenic line (BasL36), thus precluding light chain editing (*SI Appendix*, Fig. S4*A*). B cell development in these animals’ bone marrow exhibited the same hallmarks as described above for HkiL mice (Fig. 4*F*), providing additional evidence that the development of HkiL B cells was largely unaffected by light chain editing. HkiL and BasL36 mice had B cell frequencies in peripheral blood that were similar to WT and KL25H mice (Fig. 4*G* and *SI Appendix*, Fig. S4*C*), excluding extensive deletion of KL25 receptor-bearing B cells in the bone marrow or periphery (19, 20). Importantly, uniformly high levels of surface IgM on Hardy fraction E immature B cells of HkiL and BasL36 mice phenocopied nonautoreactive MD4 mice, a prototypic model of nonautoreactive B cells, which express transgenic hen egg lysozyme-specific heavy and light chains [Fig. 4*F* (41)]. We extended these studies to a second antiviral BCR. Based on the same light chain expression cassette as used for BasL36 mice, we created a vesicular stomatitis virus- (VSV-) specific light chain transgenic line, which we crossed to its matching heavy chain knock-in allele (*SI Appendix*, Fig. S4 *A* and *B* and Table S1). The resulting VI10HL mice represented, therefore, an analogous genetic set-up as the BasL36 strain, albeit with a different BCR. Importantly, the B cell development of VSV-specific VI10HL B cells in bone marrow phenocopied the one of MD4 mice (Fig. 4*F*), further supporting the conclusion that the differences in B cell development observed between mouse lines were primarily related to intrinsic properties of the antibody serving as BCR rather than to the genetic strategy used for BCR expression. Next, we directly compared the B cell development in the bone marrow of HkiL_UA_-RAG^+/–^ and HkiL-RAG^+/–^ mice (Fig. 4*H* and *SI Appendix*, Fig. S4*E*). Surface IgM levels of B220^+^IgM^+^IgD^–^ immature B cells in HkiL_UA_-RAG^+/–^ mice were significantly lower than those of HkiL-RAG^+/–^ mice and approached WT controls (Fig. 4*I*). Moreover, the immature B cell compartment of HkiL_UA_RAG^+/–^ mice was significantly larger than the one of HkiL-RAG^+/–^ mice (Fig. 4*J*), lending further support to the conclusion that the former but not the latter cells were retained at the immature B cell stage to undergo receptor editing. A relative paucity in B220^+^IgM^–^IgD^–^ pre-B cells was common to both mouse strains, differentiating them from WT controls, and was accredited to the cells’ pre-rearranged receptor allowing for pre-B cell skipping (75, 76).

B cell autoreactivity is often associated with an IgM^low^ phenotype in mature peripheral B cells (41), prompting us to extend our analysis of surface IgM levels to the B cell compartments of spleen and blood (Fig. 4 *K*–*M* and *SI Appendix*, Fig. S4 *D* and *F*–*H*). Splenic HkiL-RAG^−/−^ B cells expressed significantly less surface IgM than the average WT B cell, exhibiting levels in the range of the lower end of the WT spectrum (Fig. 4 *K* and *L*). In contrast, HkiL_UA_-RAG^−/−^ B cells expressed higher surface IgM than the average WT B cell. Analogously to splenic HkiL-RAG^−/−^ B cells also HkiL-RAG^+/+^ and BasL36 B cells in peripheral blood and spleen were IgM^low^ (Fig. 4*M* and *SI Appendix*, Fig. S4 *D*, *F*, and *I*), and in spleen exhibited a follicular B cell phenotype (*SI Appendix*, Fig. S4 *F*–*H*). Taken together, these findings suggested that HkiL and BasL36 B cells expressing the fully hypermutated KL25 BCR were less autoreactive than KL25_UA_ BCR-expressing B cells, allowing them to escape receptor editing for the most part. Downregulation of surface KL25 IgM when transitioning from the immature to the mature B cell stage may, however, indicate residual low-affinity interactions with self-antigen.

### HkiL B Cells Have a Normal Life Span and Up-Regulate Surface IgM upon Activation.

**Fig. 5.**
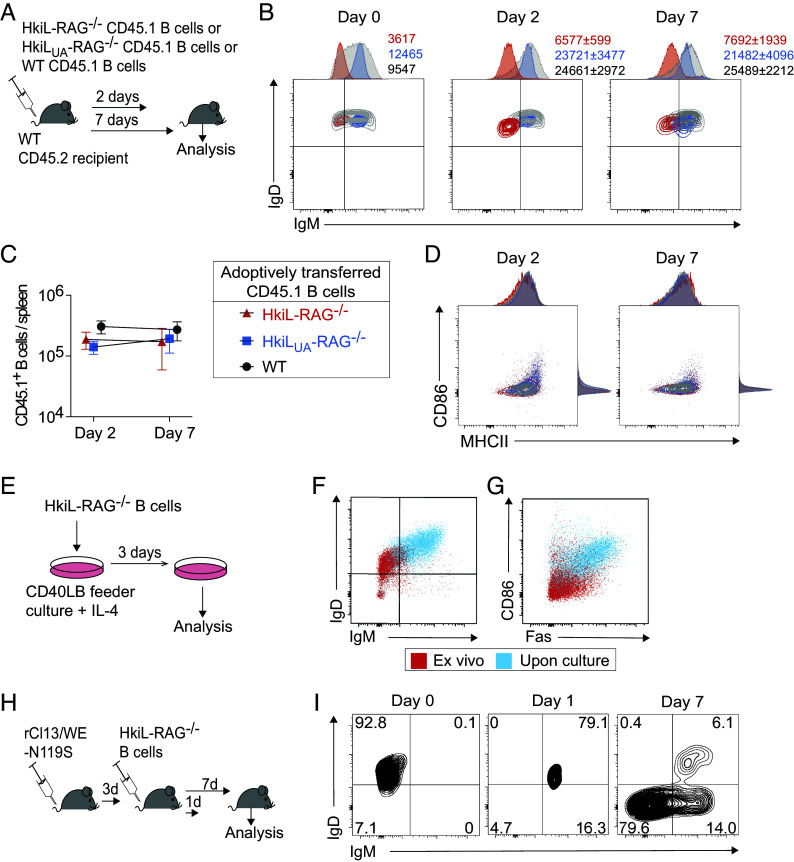
HkiL B cells have a normal half-life and up-regulate surface IgM upon activation. (*A*) Schematic of the experiment in (*B*–*D*). One-and-a-half million HkiL-RAG^−/−^, HkiL_UA_-RAG^−/−^, or WT (all CD45.1-congenic) B cells were transferred into naive WT recipients (CD45.2^+^) and were analyzed in spleen 2 and 7 d later. (*B*–*D*): IgM / IgD coexpression (*B*), cell counts (*C*), and CD86/MHC class II coexpression by adoptively transferred HkiL-RAG^−/−^ (red), HkiL_UA_-RAG^−/−^ (blue), and WT (gray) B cells (B220^+^CD19^+^CD45.1^+^ lymphocytes) prior to transfer (D0) and on d2 (n = 3 to 4) and d7 (n = 5) after transfer. One representative of two similar experiments is shown. (*E*) Schematic of the experiment in (*F* and *G*). Two million purified HkiL-RAG^−/−^ B cells were cultured for 3 d on 3T3 feeder cells expressing CD40L and BAFF [CD40LB (80)] in the presence of recombinant IL-4. (*F* and *G*) Representative FACS plot showing IgM and IgD (*F*) or CD86 and Fas (*G*) on HkiL-RAG^−/−^ B cells directly ex vivo (red) and after 4 d of activation culture (blue). n = 6 in (*F* and *G*). (*H*) Schematic of the experiment in (*I*). One million HkiL-RAG^−/−^ B cells were adoptively transferred into recipients infected with rCl13/WE-N119S 3 d earlier. Groups of animals were killed 1 and 7 d later. (*I*) IgM and IgD expression levels of HkiL-RAG^−/−^ B cells (B220^+^CD45.1^+^CD45.2^–^) prior to transfer (d0) as well as on d1 and d7. Representative FACS plots from one out of four mice in two independently conducted experiments.

Besides central deletion, which was not observed in HkiL B cells (compare Fig. 4), autoreactivity can also result in the peripheral deletion of B cells. Hence, we transferred splenic B cells from either HkiL-RAG^−/−^, HkiL_UA_-RAG^−/−^ or WT mice, all of them carrying the CD45.1 congenic marker, into syngeneic WT CD45.2 hosts and analyzed their fate 2 and 7 d later (Fig. 5*A*). HkiL B cells retained their IgM^low^ phenotype, HkiL_UA_-RAG^−/−^ cells their IgM^hi^ phenotype, differentiating them from the B cell compartment of CD45.2 recipient mice and from the adoptively transferred WT control population of CD45.1 B cells, which spanned a broad range of surface IgM levels (Fig. 5*B*). When enumerating the transferred B cells in spleen on d7, there was no clear difference between the three groups, and neither population evidenced a clear decay between d2 and d7 (Fig. 5*C*). This finding indicated HkiL and HkiL_UA_ B cells had a largely normal life span, rendering it unlikely that they were substantially affected by peripheral deletion. Importantly also, the adoptively transferred cells exhibited a resting CD86^lo^MHCII^int^ phenotype at both time points of analysis (Fig. 5*D*). To test whether IgM^lo^ HkiL B cells were responsive to in vitro activation we cultured them on feeder cells expressing CD40L and Baff [CD40LB cells (80)] in the presence of IL-4 (Fig. 5*E*). Under these stimulatory culture conditions HkiL B cells up-regulated CD86 and Fas (Fig. 5*G*) as reported for polyclonal WT B cell populations (80). Moreover, surface IgM was up-regulated (Fig. 5*F*), indicating the IgM^low^ phenotype of HkiL B cells was reversible as previously observed for autoreactive B cells (81). To test whether B cell activation by cognate antigen in vivo exerted analogous effects, we transferred HkiL cells into mice infected with the LCMV variant rCl13/WEGP-N119S (Fig. 5*H*; see below). Within 24 h after transfer into these antigen-bearing hosts, HkiL B cells up-regulated surface IgM (Fig. 5*I*). By day 7, the cells had expanded and assumed an IgM^high^ phenotype (IgD^+^ or IgD^–^) or became IgM^–^IgD^–^ double-negative, reflective of isotype class switching (Fig. 5*I* and *SI Appendix*, Fig. S5). These findings indicated the IgM^low^ phenotype of HkiL B cells was reversible and that they were not in a state of unresponsiveness that would have precluded the cells’ activation in the context of an antiviral immune response.

### HkiL B Cells Mount Potent Antibody Responses, Participate in GC Reactions, Adapt to a Viral Escape Variant, and Exhibit Partial Resistance to IFN-I Driven Decimation.

**Fig. 6.**
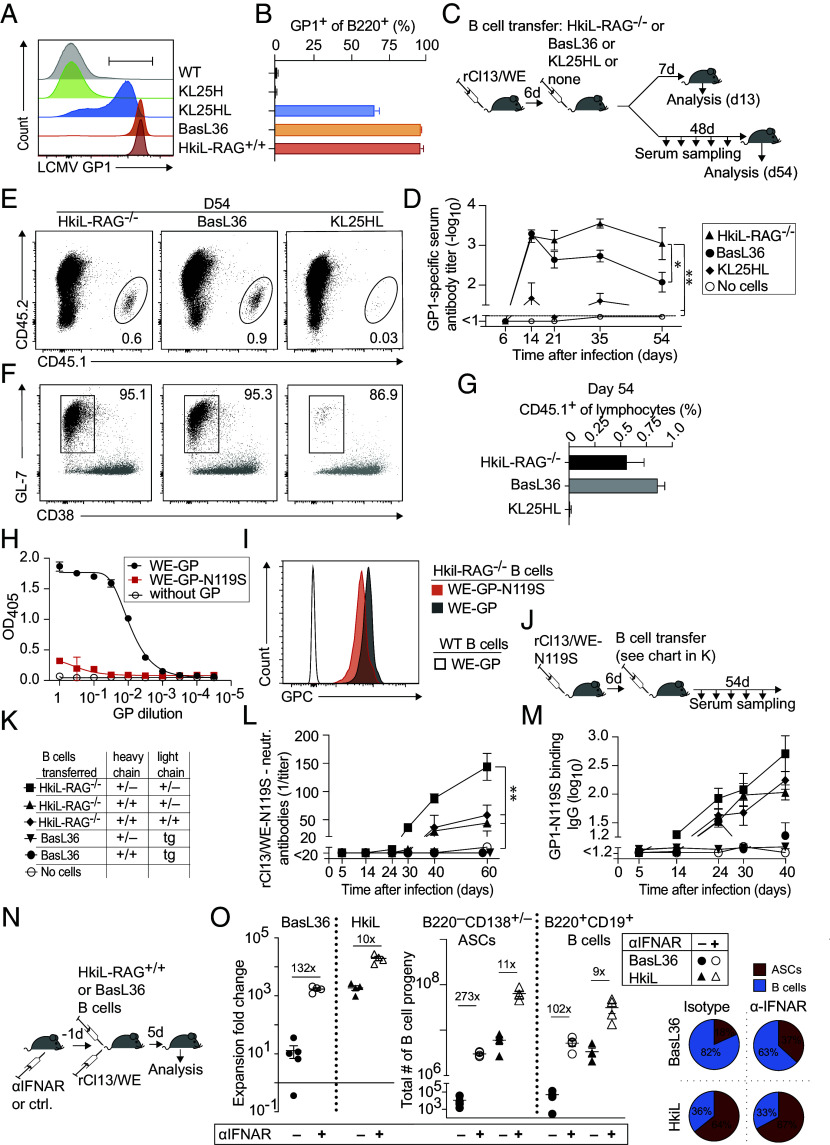
HkiL B cells mount potent antibody responses, participate in GC reactions, adapt to a viral escape variant, and exhibit partial resistance to IFN-I-driven decimation. (*A* and *B*) Representative histogram (*A*) showing the GP1-binding of splenic B cells from the indicated KL25 BCR-expressing mice (see *SI Appendix*, Fig. S4*C* for gating). The percentage of cells gated in (*A*) is reported in *B*. FACS plots in *A* show one representative out of five mice of each genotype analyzed in three experiments total (WT, BasL36, and HkiL) alongside with two KL25HL control mice, which were found as previously published (79). Bars in *B* show means ± SD. (*C*–*G*) The schematic in *C* describes the experimental design in *D*–*G*. Recipients (CD45.2^+^) were infected with rCl13/WE and 6 d later received splenic B cells from HkiL-RAG^−/−^, BasL36, or KL25HL mice. Controls were left without cell transfer. GP1-binding serum antibody titers (*D*). On d13 and d54 groups of animals were killed to analyze transferred cells in the spleen (*E*–*G*). Panels (*E* and *F*) show representative FACS plots from day 54. Panel (*E*) is gated on lymphocytes (*SI Appendix*, Fig. S6*A*); numbers in plots indicate the percentage of gated cells. Plots in *F* show transferred B cell progeny as gated in *E* in black, overlaid on polyclonal host-endogenous (CD45.2^+^) B cells in gray (see *SI Appendix*, Fig. S6*A* for gating), with numbers indicating the percentage of GL7^+^CD38^–^ GC B cells among transferred (CD45.1^+^) B cell progeny. The abundance of transferred (CD45.1^+^) B cell progeny on d54 (*G*) is expressed as the percentage of lymphocytes in the spleen. Symbols and bars in *D* and *G* show the mean±SEM of 4 mice per group. One representative of two experiments is shown. See *SI Appendix*, Fig. S6 *B* and *C* for an analysis on d13. Two-way ANOVA with Dunnett’s posttest for multiple comparisons for d14-d54 values of panel (*D*) using the HkiL recipient group as reference. *P < 0.05; **P < 0.01. (*H*) Binding of KL25 antibody to titrated concentrations of plate-bound WE-GP and WE-GP-N119S. Symbols show the means of two technical replicates. (*I*) Representative histogram showing binding of the HkiL-RAG^−/−^ B220^+^ splenocytes to WE-GP (black shaded) and WE-GP-N119S (red shaded). Splenocytes from WT mice served as technical control (transparent). (*J*–*M*) A schematic of the experimental design to (*L* and *M*) is shown in *J*. Recipient mice (CD45.2^+^) were infected with rCl13/WE-N119S and 6 d later were given splenic HkiL-RAG^−/−^, BasL36 or KL25HL B cells (CD45.1^+^). Zygosity of knock-in Ig chains or transgenic (tg) light chain of each type of donor B cell (*K*). rCl13/WE-N119S-neutralizing (*L*) and GP-N119S-binding antibody titers (*M*) were monitored. (*N* and *O*) The schematic (*N*) describes the experimental design for panel (*O*). Recipients (CD45.2^+^) were given IFN-I receptor-blocking antibody or isotype control antibody on d-1, followed by rCl13/WE infection concomitantly with transfer of 10^5^ either HkiL-RAG^+/+^ or BasL36 B cells on d0 and analysis of transferred B cell progeny in spleen 5 d later. In *O*, the expansion of adoptively transferred HkiL and BasL36 B cells was calculated (*Left*) assuming 5% splenic take (*Materials* and *Methods*). Total splenic counts of adoptively transferred (CD45.1^+^) ASCs and B cells (see *SI Appendix*, Fig. S6 *D* and *E* for gating) are shown (*Center*), and proportions of ASCs and B cells are displayed in a pie chart (*Right*). Symbols in *L* and *M* represent the mean ± SEM of 3 to 5 mice per group; symbols in *O* show individual mice. One-way ANOVA with Dunnett’s posttest in *L*. **P < 0.01. One representative experiment of two is shown.

We extended our assessment of KL25 BCR-expressing B cells in antiviral immune responses and included also the more commonly used KL25HL strain (79, 82–84) as a B cell donor. KL25HL mice carry the KL25H knock-in allele in conjunction with a transgenic KL25 light chain, thus a similar genetic configuration as the independently generated BasL36 strain, but B cells of KL25HL mice bound GP1 at lower levels and less consistently than BasL36 B cells (Fig. 6 *A* and *B*). We transferred either HkiL-RAG^-/-^, BasL36, or KL25HL B cells (all CD45.1) into 6 d rCl13/WE-infected CD45.2^+^ recipients and collected serum samples over time to determine antibody production by the transferred B cells. Moreover, we enumerated the transferred cell populations on d13 and d54 of the experiment, thus 7 and 48 d after B cell transfer, respectively (Fig. 6*C*). The GP1-binding IgG response of recipients without B cell transfer remained below detection limits throughout, validating GP1-binding antibodies as a readout of the transferred cells’ response (Fig. 6*D*). By day 14, HkiL and BasL36 B cells mounted substantial GP1-binding IgG responses, which clearly exceeded those of KL25HL B cells (Fig. 6*D*). At later time points the antibody response of HkiL B cells tended to exceed the one of BasL36 B cells, indicating the knocked-in HkiL light chain was somewhat superior to its transgenic BasL36 counterpart in terms of sustained antibody formation. All transferred B cell populations assumed a GC phenotype, yet the progeny of HkiL and BasL36 B cells were significantly more abundant than those of KL25HL B cells, which expanded within the first 8 d after transfer (*SI Appendix*, Fig. S6 *B* and *C*) but by day 54 approached the detection limit (Fig. 6 *E*–*G* and *SI Appendix*, Fig. S6*A*). The ability of GC B cells to hypermutate and improve their binding to viral escape variants represents an important feature of the antiviral B cell responses. rCl13/WE carries the WE-GP, to which KL25 binds with high affinity (67). To experimentally test the ability of HkiL B cells to hypermutate we used a genetically engineered KL25 escape variant virus (rCl13/WE-N119S) carrying a GP point mutation resulting in ~1,000-fold lower KL25 binding affinity and a complete loss of KL25 neutralizing capacity [Fig. 6*H*, (63, 67, 85)]. GPC-N119S binding to HkiL B cells was lower than WT GPC binding, as expected, but under the saturating conditions tested was still clearly detectable (Fig. 6*I*). To test for BCR adaptation we performed adoptive B cell transfer into day 6 rCl13/WE-N119S-infected mice (Fig. 6*J*). HkiL and BasL36 B cells were used side-by-side, but as a further variable we tested B cells that expressed either one or two copies of the targeted heavy and/or light chain loci (Fig. 6*K*). Recipients of HkiL cells carrying only one KL25 heavy and light chain allele each mounted a potent rCl13/WE-N119S-neutralizing antibody response, which increased continuously from day 30 to day 60 of the experiment (Fig. 6*L*). Such responses, albeit at significantly lower levels, were also detectable in recipients of HkiL cells carrying two KL25 heavy chain alleles and either one or two light chain alleles. In stark contrast, BasL36 cells failed to adapt to rCl13/WE-N119S irrespective of the cells’ homozygous or heterozygous IgH chain, indicating that adaptation of the knocked-in light chain expressed from its natural locus was key. ELISA assays determining GP1-N119S-binding antibodies evidenced an analogous hierarchy of responses, and GP1-N119S-specific antibody responses were only consistently detected in recipients of HkiL B cells (Fig. 6*M*). We and others have reported that LCMV-specific B cells undergo biased terminal differentiation into short-lived antibody-secreting cells, when triggered in the first 3 d after infection (79, 82–84). This process is driven by IFN-I-driven inflammation and results in a substantial depletion of virus-specific B cells, termed “B cell decimation”. Here we tested to which extent HkiL B cells were affected by this process. Unlike in the above experiment where antiviral B cells were transferred 3 to 6 d after infection we transferred HkiL or BasL36 B cells 1 d prior to infection and analyzed the expansion of the transferred cells 5 d after virus was administered (Fig. 6*N*). To assess the impact of IFN-I-driven B cell decimation, interferon type I receptor blocking monoclonal antibody (αIFNAR) was administered to half of the animals in each group. Both, HkiL as well as BasL36 B cells were subject to decimation as evident from higher B cell progeny numbers in αIFNAR-blocked than in isotype control antibody-treated recipients (Fig. 6*O* and *SI Appendix*, Fig. S6 *D* and *E*). However, HkiL B cells with a heavy and light chain knock-in BCR were far less affected by IFN-I-driven decimation (~10-fold) than BasL36 B cells expressing the same light chain from a transgenic locus (>100-fold). The resulting differences in B cell progeny numbers were even somewhat more pronounced for ASCs (~270-fold) than for B cells (~100-fold), and transferred HkiL B cells yielded a higher proportion of ASC progeny than BasL36 B cells irrespective of IFNAR blockade.

Taken together these experiments indicated that the IgM^low^ phenotype of HkiL and BasL36 B cells did not preclude these cells’ effective participation in antiviral immune responses, which included GC formation and long-term antibody production. The more consistent and higher levels of KL25 BCR expression on HkiL and BasL36 cells (compare Fig. 6 *A* and *B*) rendered these cells’ antiviral responses superior to the ones of KL25HL B cells. For effective affinity maturation, however, as well as for the cells’ optimal functioning in the early phase of LCMV infection, the KL25 BCR had to be expressed from the natural heavy and light chain loci.

## Discussion

The present study suggests the protective murine B cell repertoire against a naturally persisting viral intruder is centrally pruned by receptor editing. This observation proposes specific antiviral tolerance owing to viral mimicry of self, a viral strategy for the avoidance of humoral immune defense that is expected to facilitate the pathogen’s persistence. The supposed autoreactivity of the KL25 BCR is likely due to its known interactions with a specific glycan residue on the GP1 subunit, the deletion of which reduces KL25 binding to GP1 and renders the antibody non-neutralizing (63). While the binding of antibodies to sugar moieties on virion envelopes would offer an obvious solution for the immune system to overcome viral glycan shields, these sugar moieties are molecularly identical to those on host cell proteins, and the capacity of binding to such structures is expected to come at the expense of BCR autoreactivity. It, therefore, appears a difficult and potentially dangerous path to antiviral defense. Rampantly self-reactive clones, even if potentially effective against a broad range of viral intruders (86, 87), must be eliminated in the bone marrow to avoid autoimmune disease, resulting in the underrepresentation of suitable glycan-reactive B cell clones in the pre-immune repertoire. Judging from the small proportion of only ~4% GPC+GP1+ B cells in HkiL_UA_ mice with two functional RAG alleles, a very substantial proportion of GPC+GP1+ B cells in these animals are edited prior to bone marrow egress. We consider it likely that the repertoire of GPC+GP1+ B cells in WT mice is subject to analogous bottlenecks. While rare clones of GP1-reactive cells populate the periphery of mice, are responsive to antigenic challenge and contribute to antiviral protection, the paucity in suitable clones is likely to contribute to comparably weak and delayed nAb responses to persistence-prone pathogens such LCMV in mice and, perhaps similarly, to HIV and HCV in humans (1–6).

Interestingly, the phenotype of monoclonal HkiL B cells differs from HkiL_UA_-expressing B cells in two notable ways. First, the fully hypermutated KL25 receptor of HkiL B cells was not edited to any appreciable extent, which suggests the HkiL receptor is less autoreactive than the one of HkiL_UA_ B cells. We consider it most plausible that the KL25 antibody represents a so-called “redeemed” specificity, following a concept pioneered by the Goodnow laboratory (88–91). It proposes that the naive B cell repertoire is built to comprise B cells with low- to intermediate-level autoreactivity, in order to allow for a better coverage of a vast diversity of potential microbial invaders. Redemption occurs upon activation of autoreactive B cells, which hypermutate their receptor and are selected for reduced autoreactivity while preserving or improving their reactivity to the pathogen. The KL25 B cell hybridoma was originally generated from a mouse that had been hyperimmunized with LCMV (64). Based on the present data, we suspect that the naive precursor of the KL25 B cell exhibited significant autoreactivity. During affinity maturation, however, it may not only have been selected for improved binding to LCMV-GP1 but may have been redeemed, too, reducing its binding to molecular self-structures.

The second notable difference between HkiL_UA_ and HkiL B cells consists in the latter but not the former cells down-regulating surface IgM at the mature B cell stage. The IgM^lo^ phenotype of HkiL B cells was recapitulated in BasL36 mice and thus was linked to the hypermutated KL25 light chain. At first glance, the IgM^hi^ phenotype of HkiL_UA_ B cells may seem at odds with the proposal that IgM is down-regulated when autoreactive B cells escape central deletion (91). The experience with several autoreactive BCRs across different models shows, however, that the extent of IgM downregulation varies considerably between individual autoreactive BCRs (92–95). Accordingly, it remains to be investigated whether more pronounced IgM downregulation by HkiL as compared to HkiL_UA_ B cells represents an intrinsic structural feature of these cells’ respective BCRs and/or whether it relates to the characteristics of interaction with a yet undefined self-ligand(s) (96).

One conclusion of practical importance that can be derived from this study relates to the advantages of BCR-engineered cells with a receptor expressed entirely from V(D)J knock-ins as compared to randomly inserted transgenes. As our study shows, the efficient hypermutation of B cells for adaptation to viral escape variants can require that heavy and light chains are both expressed from their respective autologous genetic loci. This finding suggests that hypermutation not only of the heavy chain but also of the light chain contributed essentially to affinity maturation. Moreover, HkiL B cell adaptation to the GP-N119S variant was most efficient when only one copy of each allele was present. The reason for the latter likely consists in the “dilutive” effect of the second rearranged allele (29–31), precluding the efficient selection of clones, which by means of hypermutation have succeeded in adapting one of their two alleles. Besides these effects on affinity maturation, the heightened resistance of HkiL B cells to IFN-I-driven decimation highlights the importance of physiological BCR expression for studies on B cell infection biology. Finally, the direct comparison of HkiL, BasL36, and KL25HL B cells expressing the same KL25 light chain either as knock-in or as transgene, respectively, has evidenced differences in the cells’ capacity for sustained high-titer antibody production. Several not mutually exclusive explanations may apply. First, differences in concatemeric copy numbers and/or genomic insertion sites of transgene cassettes are likely to account for differences such as those observed between KL25HL and BasL36 B cells. Second, the superiority of HkiL VJ knock-in B cells may be due to the long-range genomic interactions in the kappa chain locus (reviewed in ref. 97): Like other commonly used light chain expression cassettes (19, 41), those carried by KL25HL and BasL36 B cells comprise the intronic kappa enhancer (iEκ) as well as the 3′ kappa enhancer (3′Eκ) located ~9 kb downstream of the Cκ gene but they lack the distal kappa enhancer (dEκ) situated another ~8 kb downstream. Neither can transgene cassettes recreate genomic interactions such as those established by iEκ and 3′Eκ with sequences 24 kb downstream of dEκ (98).

An obvious limitation of our study consists in its focus on the prototypic LCMV-nAb KL25. It remains possible that other molecular solutions for LCMV GP1-binding can be found, avoiding the autoreactive challenges discussed above. We acknowledge further that the autoreactivity of the KL25_UA_ antibody can only be inferred based on the corresponding B cells’ extensive receptor editing. A formal demonstration of autoreactivity would require the identification of specific antibody targets in the mouse proteome, which may be difficult if not impossible in case cross-reactivity to self was indeed based on glycan structures as discussed above. Finally, we are aware that BCR hypermutation can also be a source of B cell autoreactivity rather than redemption (99–103). Accordingly, it remains to be formally tested whether hypermutations in the heavy chain of the KL25_UA_ receptor contribute to or may even be required for autoreactivity of this BCR. The less autoreactive behavior of the HkiL BCR, consisting of the same heavy chain in conjunction with the fully hypermutated KL25 light chain, suggests light chain hypermutations lessened KL25 autoreactivity. It does not, however, exclude the possibility that heavy chain hypermutations may exert opposing effects.

In summary, our findings in a physiological setting of virus–host relationship suggest that persistence-prone viruses have developed surface structures that molecularly resemble self, confronting the immune system with the daunting task of preserving self-tolerance in the fight against a self-mimicking infectious intruder. B cell tolerance and redemption mechanisms are predicted to shape this process, defining the limitations of such antibody responses in terms of speed and vigor.

## Materials and Methods

Materials and Methods are provided as a part of *SI Appendix* and detail mice and animal experimentation, adoptive B cell transfer, virus infection and monoclonal antibody treatment, single guide RNA (sgRNA) identification and testing, pronuclear injection and targeting constructs, long-range PCR for Ig kappa VJ knock-in validation as well as KL25H light chain sequencing. Information on recombinant viral protein and antibody production, flow cytometry, viruses, virus titration, virus neutralization assays, and cell lines is supplied, together with methodology for enzyme-linked immunosorbent assays, B cell culture, statistical analysis, and principles of materials sharing.

## Supplementary Material

Appendix 01 (PDF)

## Data Availability

Raw data reported in this study are deposited (104) in Zenodo under the accession DOI: 10.5281/zenodo.8408593.
