# Supplementary material for: Central tolerance shapes the neutralizing B cell repertoire against a persisting virus in its natural host
Source: Proc Natl Acad Sci U S A. 2024 Mar 6;121(11):e2318657121. doi: 10.1073/pnas.2318657121 (PMC10945855; doi:10.1073/pnas.2318657121)
Supplement: Supplementary file 1 — Appendix 01 (PDF) [file pnas.2318657121.sapp.pdf]

Supplementary Appendix to Florova, Abreu-Mota et al.

Methods

Mice and animal experimentation

C57BL/6 wt mice were originally purchased from Charles River laboratories and were bred locally. KL25H mice (B6-IgH(VDJ-KL25)^{Zbz}) carry an immunoglobulin heavy chain (HC) VDJ knock-in (KI) derived from the LCMV-neutralizing monoclonal antibody-producing hybridoma KL25 (1) and express the CD45.1 congenic marker. In KL25HL (B6-IgH(VDJ-KL25)^{Zbz} x B6J-Tg(KL25L)^{Tac}) and Basl36 mice (B6-IgH(VDJ-KL25)^{Zbz} x B6-Tg(BasL25)^{Ctmb}) the KL25H HC KI is paired with the matching KL25 light chain (LC), expressed as a randomly integrated transgene (2, 3). KL25H-RAG$^{-/-}$ mice were obtained by intercrossing KL25H mice with RAG2-deficient B6.Cg-*Rag2*$^{tm1.1Cgn}$/J mice (4). HkiLUA-RAG$^{-/-}$ and HkiL-RAG$^{-/-}$ mice were generated by CRISPR/Cas9-based VJ knock-in (see below) into the Ig kappa locus of KL25H-RAG$^{+/-}$ and KL25H-RAG$^{-/-}$ mice, respectively, and were further crossed to KL25H-RAG$^{-/-}$ mice. RAG2-sufficient HkiLUA (B6-IgH(VDJ-KL25)^{Zbz} x B6-IgL(VJ-KL25uca)^{Dpin}) and HkiL mice (B6-IgH(VDJ-KL25)^{Zbz} x B6-IgL(VJ-KL25)^{Dpin}) were generated by intercrossing HkiL-RAG$^{-/-}$ mice with CD45.1-congenic C57BL/6 mice. To obtain HkiL-RAG$^{-/-}$ mice with hemizygous HC and LC KI loci for adoptive transfer experiments, HkiL-RAG$^{-/-}$ mice were intercrossed with CD45.1-congenic RAG2$^{-/-}$ mice. MD4 mice (C57BL/6-Tg(IghelMD4)4Ccg/J), transgenic for HC and LC of the anti-hen egg lysozyme antibody HyHEL10 have been described (5). VI10 mice (B6-IgH(VDJ-VI10)^{Zbz} x B6-Tg(VI10L)^{Dpin}) carry an immunoglobulin HC VDJ KI derived from the VSV glycoprotein-specific monoclonal antibody-producing hybridoma VI10 (1), which is paired with a matching, randomly integrated transgenic VI10 LC transgene (first described in this manuscript). TgL mice express the transgenic KL25 light chain of KL25HL mice without the matching heavy chain (2, 3, 6). All mice were kept under specific-pathogen-free conditions for colony maintenance and experiments. Experimental groups were sex and age-matched. Animals of both sexes were used and were generally between 8-16 weeks old. Mice were bred at the Laboratory Animal Sciences Center (LASC) of the University of Zurich and at the ETH Phenomics Center Zurich (EPIC). Experiments were performed at the University of Basel in accordance with the Swiss law for animal protection and with permission by the Cantonal Veterinary Office of Basel City (No. 28403/2654, 31652/2654, 35149/2654).

Adoptive B cell transfer, virus infection and monoclonal antibody treatment

B cells in the experiment to Fig. 2F-G were FACS-sorted based on the gating strategy outlined in Fig. S2A and 10,000 of each subset were adoptively transferred into recipients by the intravenous (i.v.) route. For the experiments in Figs. 5 and 6, splenic B cells were purified using the EasySep mouse B cell isolation kit (STEMCELL). 1’500’000 and 5’000 cells were injected i.v. into recipients for the experiments in Fig. 5 and Fig. 6, respectively. Assuming an approximate take of 5% (7-9), the above numbers of cells transferred correspond to approximately 500, 75,000 and 250 engrafted cells per spleen in the experiments to Fig. 2, Fig. 5 and Fig. 6, respectively. TgL mice were used as recipients in all adoptive B cell transfer experiments as described (2, 3, 6) to avoid anti-idiotypic rejection of transferred B cells. Mice were infected with 2-6x10^6 PFU

of LCMV i.v.. 1 mg of IFNAR-blocking antibody (MAR1-5A3, BioXCell) or of MOPC-21 mouse IgG1 isotype control antibody (BioXCell) was administered intraperitoneally (i.p.) on day -1 of LCMV infection.

sgRNA identification and testing

CrispRGold (https://crisprgold.mdc-berlin.de/) was used to identify sgRNAs that target genomic regions flanking the $J_\kappa 1$-$J_\kappa 5$ exons in the intronic region but were not included in the homology arms of the targeting construct. Two such sgRNAs for 5' and 3' cutting sites (crRNA51: AAGCATGCGTGGAAGCGCTT, crRNA52: CAAGCATGCGTGGAAGCGCT, crRNA31: GGGTCTGACTGCAGGTAGCG, crRNA32: TGAGATCTGGGTCTGACTGC) were chosen based on proximity to the respective homology arms, they were synthesized (IDT) and tested in vitro for cutting efficiency on PCR amplicons covering the respective sequence stretches. Based on performance in these assays, crRNA52 and crRNA32 were used for pronuclear injections. PCR reactions were performed to generate genomic DNA fragments flanking the Ig kappa J regions for sgRNA testing. PCR products were purified using the Qiagen PCR purification kit and diluted to a 50 ng/ul concentration. Equimolar ratios of crRNA and tracrRNA (IDT) were mixed for a final concentration of 100 µM, heated at 95°C for 5 min. and cooled to room temperature. RNP complexes were created by mixing 1µl (100 pmol) of sgRNA and 1.6 µl (100 pmol) of *S. pyogenes* Cas9 protein (IDT) in IDT buffer. The coupling reaction was allowed to proceed for 30 min. at RT. Subsequently, 400ng (8 µl) of DNA substrate was added per reaction and filled up with IDT buffer to a total of 50 µl. These Cas9 digestion reactions were incubated for 60 min. at 37°C. Analogous reactions without Cas9 protein served as controls. For analysis of the digestion reaction, the resulting fragments were separated by agarose gel electrophoresis and visualized by Ethidium bromide staining.

Pronuclear injection and targeting constructs

Targeting vectors for the generation of the HkiLUA and HkiL light chain knock-in mice was generated based on the pVKR2neo plasmid (10), which was generously provided by Dr. D. Nemazee (The Scripps Research Institute, La Jolla, CA, USA) and was modified by removing both the neomycin resistance and the thymidine kinase cassettes. Pronuclear injections were performed at the Institute of Laboratory Animal Science (LTK) of the University of Zurich, at the ETH Phenomics Center Zurich (EPIC) and at the Centre for Transgenic Models (CTM) of the University of Basel. Founder animals were bred under specific pathogen-free conditions (SPF). To prepare the pronuclear injection mix, 10 pmol of *S. pyogenes* Cas9 protein (IDT), 18.4 pmol of each crRNA and tracrRNA (IDT) were mixed with 10x injection buffer (100mM Tris-HCL, 1mM EDTA (pH8.0)) and incubated at 37°C for 10 min. Subsequently, 1 µg of plasmid DNA was added and the reaction volume was adjusted to 50 µl by the addition of double-distilled water. Fertilized oocytes were injected and after a brief incubation period were implanted into the pseudo-pregnant foster mothers.

Long-range PCR for Ig kappa VJ knock-in validation

Phusion polymerase (NEB) was used for long-range PCR to confirm correct integration of the KL25 and $KL25^{UA}$ light chain VJ elements into the mouse kappa locus, respectively. Primer pairs targeted the inserted VJ and regions outside of the 5' and 3' homology arms, respectively: 5HA-fwd: GCA AAT GTC TGA TGA GTG CTT GTC A, 5HA-rev: GGC TTC TGC TGG TAC CAG ATC ATG TA, 3HA-fwd: GCT TCC TGC TAA TCA GTG CCT CAG GTA A, 3HA-rev: TTG AGC TCT GGA AGG CAG AAT AGT AGA AGG C. The

following cycling conditions were used: 98°C for 1 min.; 35× (98°C for 10 s, 69°C for 30 s, and 72°C for 2 min.); 72°C for 10 min. Genomic mouse DNA was purified using the DNA Purification Kit (Qiagen), and 50 ng were used for each reaction.

Recombinant viral protein and antibody production

The LCMV-WE GP1 gene fragment (comprising residues 81 to 241) was cloned into the pHLsec vector for transient expression as a secreted, hexahistidine-tagged protein in HEK293 cells as described (11). Five days after transfection, the expression medium was collected, supplemented with NaCl (to a final concentration of 0.75M) and Tris pH 8.0 (15mM), clarified (10,000 x g, 20 min., 4˚C), and brought over a 5-ml HisTrap excel column (Cytiva). Protein was eluted using a 15-750 mM imidazole gradient in 10 mM Tris pH 8.0, 200 mM NaCl, and further purified by size-exclusion chromatography over a superdex 200 increase 10/300 GL column (Cytiva) using 15 mM Hepes pH 7.4, 150 mM NaCl as running buffer. Labeling with Alexa Fluor™ 647 (AF647) was carried out with a kit from Thermo Fisher Scientific following the manufacturer's instructions. The LCMV-WE GPC ectodomain (comprising residues 1- 431, which includes the protein's signal peptide) with a GP1-GP2 cleavage site mutation and a Twin-Strep-tag fused to its C-terminus (12) was produced by transient transfection in CHO cells at the Protein Expression Core Facility (PECF) of the Swiss Federal Technical Highschool Lausanne, Switzerland (EPFL). The N119S mutant version thereof was produced analogously. Protein-containing supernatants were directly used for flow cytometric staining and ELISA.

Flow cytometry

Organs were collected and cell suspensions prepared in B cell medium (DMEM supplemented with 10% FCS, HEPES, sodium pyruvate and non-essential amino acids). Splenic single cell suspensions were prepared using a syringe plunger and a metal mesh in a Petri dish, and subsequently pipetting up and down. For collection of bone marrow, bones were flushed with a syringe and needle, and cells were dispersed by pipetting up and down. The peritoneal cavity was lavaged using a medium-containing syringe and needle. Dead cells were excluded from the analyses using the Zombie UV Viability kit (BioLegend). For the identification of HkiL-RAG$^{-/-}$ founders, the following antibodies were used: αCD3-PE (500A2, BD Biosciences, αCD19-FITC (1D3, Biolegend), αB220-PE-Cy7 (RA3-6B2, BD Biosciences). LCMV-GP-specific B cells were identified using fluorescently labeled recombinant GP1 and/or GPC proteins as follows. Cells were incubated on a shaker at 37ºC for 30 min. in 200 µl GPC-StreptagII-containing transfection culture supernatant to which 5 µg of Fc receptor-blocking antibody (2.4G2, BioXcell) and 0.3 µg of GP1-AF647 was added. Samples were subsequently washed and resuspended in 50 µl FACS buffer containing Streptactin-PE (IBA) and anti-B220-BV421(RA3-6B2 BioLegend), anti-IgM-PerCP-eFluor710 (II/41, eBioscience), anti-IgD-APC-Cy7 (11-26c.2a, BioLegend). For characterization of HkiL$_{UA}$ and HkiL B cells the following antibody combination was used: 5 µg Fc receptor-blocking antibody (2.4G2, BioXcell), anti-CD3-BV421 (BD, 17A2) or anti-CD3-FITC (BD, 145-2C11), anti-B220-BV605 (BioLegend, RA3-6B2) or anti-B220-BV421 (BioLegend, RA3-6B2), anti-CD21-PerCP-Cy5.5/BV711 (BioLegend,7E9), anti-CD23 -PE/PE-dazzle594 (BD/biolegend, B3B4), anti-CD24 – FITC (BD, M1/69), anti-IgM-APC (BioLegend, RMM-1) or anti-IgM-PerCP-eFluor710 (eBioscience, II/41), anti-IgD-APC-Cy7 (BioLegend, 11-26c.2a), anti-CD19-PE (BioLegend, 1D3), anti-CD11b-APC-Cy7 (BioLegend, M1/70), anti-CD5-PE (BD, 53-7.3), anti-CD19-APC (BioLegend, 6D5), anti-CD95 (Fas) -APC

(BioLegend, SA367H8), anti-CD86-PE (BioLegend, A17199A), anti-kappa (BD biosciences, 187.1), anti-lambda (BD biosciences, R26-46), Anti-CD117-APC (Biolegend, 2B8), CD43-PE/APC (BD, S7 or Biolegend, S11. Anti-Kappa antibody was incubated in a separate step for 10 min. at room temperature to avoid cross-labeling with other antibodies in the staining mix. Samples were washed and the remaining antibodies were added in a master mix. Staining was performed for 15 min. at room temperature before washing and acquisition. Cell viability was assessed with either 4′,6-diamidino-2-phenylindole (Sigma-Aldrich) when live cell acquisition was performed or Zombie UV Fixable viability dye (Biolegend). In adoptive transfer experiments spleens of mice were collected after sacrifice and single cell suspensions were prepared. One tenth of each spleen was used for staining. Splenocytes were incubated with GPC-Streptag protein and 5 µg of Fc receptor blocking antibody at 37°C for 30 min. on a shaker. After a wash step 50 µl of staining solution was added consisting of the following staining reagents in FACS buffer: Streptactin-APC (IBA), anti-CD45.2-BV605 (BioLegend, 104), anti-CD45.1-BV421 (BioLegend, A20), anti-B220-AF700 (BioLegend, RA3-6B2), anti-IgM-PerCP-eFluor710 (eBioscience, II/41), anti-GL-7-AF488 (BioLegend, GL7), anti-CD38-PE-Cy7 (BioLegend, 90) and anti-CD138-PE (BD, 281-2). Samples were measured on either an LSRFortessa (Becton Dickinson) or on an Aurora flow cytometer (Cytek) and data were analyzed using FlowJo software (BD biosciences). Splenocytes were counted using the CTL-LDC kit on an CTL S6 Ultimate CTL counter (Immunospot). VSVG-binding B cells were detected as previously described (2).

Viruses, virus titration, virus neutralization assays and cells lines

Engineered LCMV strain Clone 13 (rCl13/WE) expressing the glycoprotein of the LCMV strain WE (WE-GP) and an otherwise identical variant virus carrying the KL25 escape mutation N119S in WE-GP (WE-GP-N119S; rCl13/WE-N119S) were used (12). The viruses were propagated on BHK21 cells (ECAAC). NIH 3T3 (ATCC) cells were used for titration in immunofocus assays (13). Stably transfected fibroblasts expressing BAFF and CD40L were used as feeders for in vitro B cell activation (14). Immunofocus reduction neutralization tests were performed to determine neutralizing antibody titers in the serum of mice and to assess the neutralizing activity of monoclonal antibodies. NIH 3T3 fibroblasts served as a cell substrate in these assays. Neutralizing titers of sera were determined as the highest dilution yielding at least 50% reduction in the number of infectious foci.

Enzyme-linked immunosorbent assay (ELISA)

GP1-binding antibodies in mouse serum, and binding of recombinant antibodies sequenced from TgH mouse were determined using recombinant HEK293 cell-derived GP1-Fc fusion protein as a substrate following established protocols (6, 15). Briefly, 96-well high binding plates (Greiner Bio-One) were coated with 0.7 µg/ml goat anti-human IgG Fcγ antibody (Jackson Immunoresearch, 109-005-098) in coating buffer (Na_2CO_3 15mM, $NaHCO_3$ 35mM, pH9.6) at 4°C overnight. Then, the plates were blocked for 2 hours at room temperature with PBS / 0.05% Tween / 5% milk (also used as buffer in the subsequent steps). GP1-Fc was added and incubated for 1h, followed by three wash steps with PBS / 0.05% Tween (PBS-T). Serum samples or monoclonal antibodies were added in a serially diluted manner and were incubated for 1h at RT. Following three more PBS-T washing steps goat anti-mouse IgG-HRP conjugate antibody (1:750, Jackson Immunoresearch) was added. Excess secondary antibody was washed away and HRP activity was detected using ABTS as a chromogen (Pierce). The capacity of KL25 antibody to wildtype GP or mutated GP-N119S was determined using a HEK293-derived extracellular WE-GP

domain with a C-terminal StreptagII sequence (GP-Streptag (12)) as an ELISA substrate. The ELISA assay was performed analogously to the above, except that GP-Streptag was captured by coating ELISA plates with 0.5ug/ml of Strep-TactinXT (Iba), and that the plates were blocked with 0.2% BSA in PBS. OD_{405} was determined in an ELISA reader.

For antibody titer assessments in mouse serum we performed serial dilutions to determine the minimal concentration at which a signal ≥2-fold technical background was reached. Alternatively, a curve fit approach with GEN5 software (BioTek Instruments) was used, notably when assessing the binding behavior of monoclonal antibodies.

B cell culture

For B cell activation cultures, CD40LB cells were seeded in T25 flasks (TPP) at a density of 10^5 cells per cm^2 and their proliferation was stopped by gamma irradiation (30 Gy). Subsequently, the culture medium was replaced with 2.5ml fresh B cell medium consisting of DMEM (Sigma) supplied with 10% FCS (Life Biosciences), 2 mM l-glutamine, 2 mM sodium pyruvate, 2 mM Hepes (Gibco), non-essential amino acids (Gibco), β-mercaptoethanol (Sigma), and 10 µg/mL gentamicin (Lonza)), and 12h later $5x10^6$ freshly isolated B cells were added. The co-culture was supplemented with 1 ng/ml IL-4 (BioLegend) and incubated at 37°C for 48 hours. CD40LB fibroblasts (14) were provided by Dr. S. Reddy (ETH Zurich, Basel, Switzerland) under a material transfer agreement and authorization by Dr. D. Kitamura (Tokyo University of Science, Tokyo, Japan). They were passaged in DMEM 10% FCS after the initial one-week passage in selection media containing 2.5ug/ml Puromycin and 0.5mg/ml G418

KL25H light chain sequencing

The KL25H B cell subset binding to LCMV-GPC and -GP1 was FACS-sorted and RNA was extracted and transcribed to cDNA. Rearranged light chain genes were amplified using multiple individual PCRs with a degenerate forward primer set binding to the variable domain signal peptide region and reverse primers in the light chain kappa or lambda constant region. A second round of PCR was conducted on purified PCR fragments (Qiagen PCR purification kit) using analogous primer sets that were extended by restriction sites for subsequent cloning of amplified fragments into expression vectors (Mouse IgG Library primer set, Progen). Upon transformation into competent bacteria, individual colonies were Sanger-sequenced and functional VJ sequences were identified using IgBlast. For recombinant expression, the obtained sequences were synthesized (Genescript) and introduced into light chain expression cassettes for recombinant expression in conjunction with the KL25 heavy chain in a mouse IgG2a format as described (16).

Statistical Analysis

The GraphPad Prism software version 9 (GraphPad Software) was used for all statistical analyses. For comparison of one parameter between multiple groups one-way analysis of variance (ANOVA) was performed and for comparison of multiple parameters between two or more groups two-way ANOVA was used, both followed by Tukey's post-test for multiple comparison. For statistical analysis of absolute cell counts, values were log-converted to obtain a near-normal distribution. *P* values ≥0.05 were considered as not statistically

significant. Binding and neutralization curves of monoclonal antibodies were fitted using GraphPad Prism software 7 (GraphPad Software).

Materials sharing

Material transfer agreements with standard academic terms will be established to document reagent sharing by the corresponding author's institution.

Supplementary References

1. L. Hangartner *et al.*, Antiviral immune responses in gene-targeted mice expressing the immunoglobulin heavy chain of virus-neutralizing antibodies. *Proc Natl Acad Sci U S A* **100**, 12883-12888 (2003).
2. B. Fallet *et al.*, Interferon-driven deletion of antiviral B cells at the onset of chronic infection *Sci Immunol* **1**, eaah6817 (2016).
3. K. Narr *et al.*, Vaccine-elicited CD4 T cells prevent the deletion of antiviral B cells in chronic infection. *Proc Natl Acad Sci U S A* **118** (2021).
4. Z. Hao, K. Rajewsky, Homeostasis of peripheral B cells in the absence of B cell influx from the bone marrow. *J Exp Med* **194**, 1151-1164 (2001).
5. C. C. Goodnow *et al.*, Altered immunoglobulin expression and functional silencing of self-reactive B lymphocytes in transgenic mice. *Nature* **334**, 676-682 (1988).
6. B. Fallet *et al.*, Chronic Viral Infection Promotes Efficient Germinal Center B Cell Responses. *Cell Rep* **30**, 1013-1026 e1017 (2020).
7. P. Dosenovic *et al.*, Anti-HIV-1 B cell responses are dependent on B cell precursor frequency and antigen-binding affinity. *Proc Natl Acad Sci U S A* **115**, 4743-4748 (2018).
8. R. K. Abbott *et al.*, Precursor Frequency and Affinity Determine B Cell Competitive Fitness in Germinal Centers, Tested with Germline-Targeting HIV Vaccine Immunogens. *Immunity* **48**, 133-146 e136 (2018).
9. J. J. Taylor, K. A. Pape, H. R. Steach, M. K. Jenkins, Humoral immunity. Apoptosis and antigen affinity limit effector cell differentiation of a single naive B cell. *Science* **347**, 784-787 (2015).
10. R. Pelanda, S. Schaal, R. M. Torres, K. Rajewsky, A prematurely expressed Ig(kappa) transgene, but not V(kappa)J(kappa) gene segment targeted into the Ig(kappa) locus, can rescue B cell development in lambda5-deficient mice. *Immunity* **5**, 229-239 (1996).
11. A. R. Aricescu *et al.*, Eukaryotic expression: developments for structural proteomics. *Acta Crystallogr D Biol Crystallogr* **62**, 1114-1124 (2006).
12. R. Sommerstein *et al.*, Arenavirus Glycan Shield Promotes Neutralizing Antibody Evasion and Protracted Infection. *PLoS Pathog* **11**, e1005276 (2015).
13. M. Battegay *et al.*, Quantification of lymphocytic choriomeningitis virus with an immunological focus assay in 24- or 96-well plates. *J Virol Methods* **33**, 191-198 (1991).
14. T. Nojima *et al.*, In-vitro derived germinal centre B cells differentially generate memory B or plasma cells in vivo. *Nat Commun* **2**, 465 (2011).
15. B. Eschli *et al.*, Early antibodies specific for the neutralizing epitope on the receptor binding subunit of the lymphocytic choriomeningitis virus glycoprotein fail to neutralize the virus. *J Virol* **81**, 11650-11657 (2007).
16. M. Sahin *et al.*, Antibody bivalency improves antiviral efficacy by inhibiting virion release independently of Fc gamma receptors. *Cell Rep* **38**, 110303 (2022).
17. S. Sammicheli *et al.*, Inflammatory monocytes hinder antiviral B cell responses. *Sci Immunol* **1** (2016).

18. E. A. Moseman, T. Wu, J. C. de la Torre, P. L. Schwartzberg, D. B. McGavern, Type I interferon suppresses virus-specific B cell responses by modulating CD8(+) T cell differentiation. *Sci Immunol* **1** (2016).

Table SI. BCR knock-in and -transgenic mouse models used in this manuscript

**Mouse line**[1]	**KL25H**	**KL25HL**	**BasL36**	**HkiL**	**HkiL$_{UA}$**	**VI10**	**MD4**
**Heavy chain**[2]	knock-in	knock-in	knock-in	knock-in	knock-in	knock-in	transgenic
**Light chain**[3]	-	transgenic	transgenic	knock-in	knock-in	transgenic	transgenic
**Introduced**[4] **immunoglobulin**	KL25 heavy chain	KL25 heavy and light chain	KL25 heavy and light chain	KL25 heavy and light chain	KL25 heavy chain and unmutated ancestor light chain	VI10 heavy and light chain	HyHEL-10 heavy and light chain
**Original publication of heavy chain allele**[5]	Hangartner et al., 2003 (1)	Hangartner et al., 2003 (1)	Hangartner et al., 2003 (1)	Hangartner et al., 2003 (1)	Hangartner et al., 2003 (1)	Hangartner et al., 2003 (1)	Goodnow CC et al., 1988 (5)
**Original publication of light chain allele**[6]	Not applicable[7]	Fallet et al., 2016 (2) Sammicheli et al., 2016 (17) Moseman et al., 2016 (18)	Narr et al., 2021 (3)	This manuscript	This manuscript	This manuscript	Goodnow CC et al., 1988 (5)

[1] Mouse strain designation as used in this manuscript

[2] Pre-rearranged heavy chain expressed, either transgenic or knock-in

[3] Pre-rearranged light chain expressed, either transgenic or knock-in

[4] Antibody clone from which pre-rearranged heavy chain and/or light chain are derived

[5] Original publication where the pre-rearranged heavy chain allele has been reported

[6] Original publication where the pre-rearranged light chain allele has been reported

[7] The KL25H mouse line does not carry any pre-rearranged light chain allele

Supplementary Figures and Legends

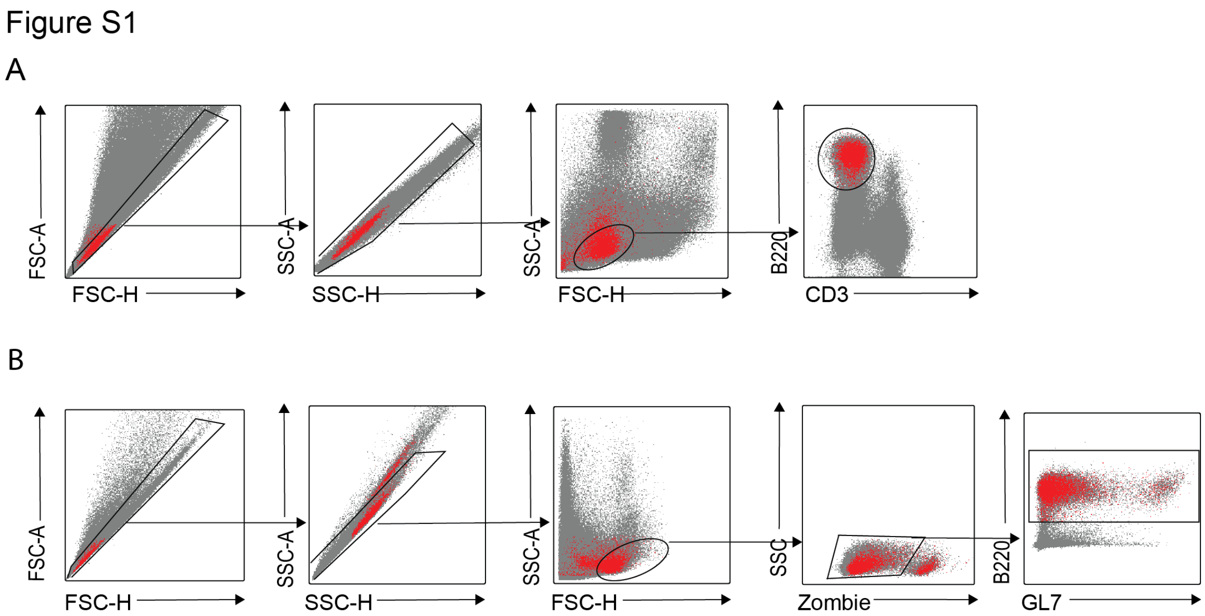

Figure S1: Flow cytometric gating strategy for the analysis of splenic B cells.

A: Gating strategy for the identification of B220^{+} splenocytes as used e.g. in the experiment to Figure 1B.

B: Gating strategy for the identification of B220^{+} splenocytes as used e.g. in the experiment to Figure 1C,D.

Figure S2

A

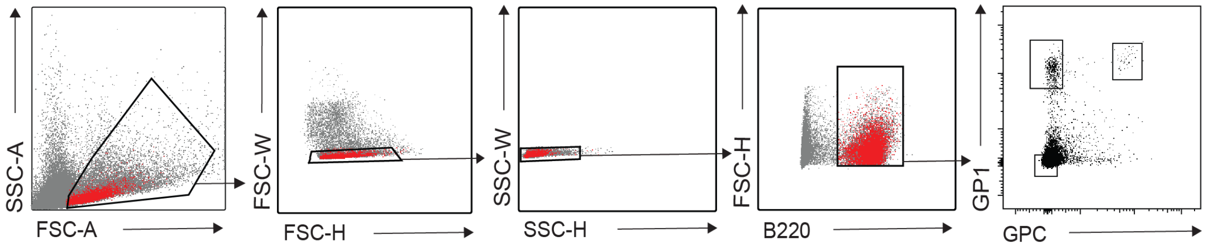

B

KL25

```
1        10        20        30        40        50        60        70        80        90        100
|        |         |         |         |         |         |         |         |         |         |
<-----------FR1---------><-1-><------FR2------><2><--------------FR3-----------------><--CDR3-><--FR4--->

QVVLTQSPAIMSASPGEKVTLTCSASSSITYMIWYQQKPGSSPRLLIYDTSNLAFGVPVRFRGSGSGTSYSLTISRMEAEDAATYYCQHWSGYPFTFGSGTKLEIK
```

IGKV4-55/IGKJ4 (KL25UA)

```
1        10        20        30        40        50        60        70        80        90        100
|        |         |         |         |         |         |         |         |         |         |
<-----------FR1---------><-1-><------FR2------><2><--------------FR3-----------------><--CDR3-><--FR4--->

QIVLTQSPAIMSASPGEKVTMTCSASSSVSYMYWYQQKPGSSPRLLIYDTSNLASGVPVRFSGSGSGTSYSLTISRMEAEDAATYYCQQWSSYPFTFGSGTKLEIK
```

IGKV14-111/IGKJ2

```
1        10        20        30        40        50        60        70        80        90        100
|        |         |         |         |         |   CDR   |         |         |         |         |
<-----------FR1---------><CDR1><-------FR2-----><2><----------------FR3---------------><--CDR3-><--FR4--->

DIKMTQSPSSMYASLGDRVTITCKASQDINSYLSWFQQKPGKSPKTLINRANRLVDGVPSRFSGSGSGQDYSLTISSLEYEDMGSYYCLQYDEFPYTFGGGTKLEIK
```

IGKV02-137/IGKJ4

```
1        10        20        30        40        50        60        70        80        90        100       110
|        |         |         |         |         |   CDR
<---------FR1-----------><---CDR1--><------FR2------><2><----------------FR3---------------><--CDR3-><--FR4--->

DIVMTQAAPSVPVTPGESVSISCRSSKSLLHSNGNTYLYWFLQRPGQSPQLLIYRMSNLASGVPDRFSGSGSGTAFTLRISRVEAEDVGVYYCMQHLEYPFTFGSGTKLEIK
```

IGKV10-96/IGKJ2

```
1        10        20        30        40        50        60        70        80        90        100
|        |         |         |         |         |   CDR
<---------FR1----------><CDR1><------FR2------><2><--------------FR3-----------------><-CDR3-><---FR4-->

IQMTQTTSSLSASLGDRVTISCRASQDISNYLNWYQQKPDGTVKLLIYYTSRLHSGVPSRFSGSGSGTDYSLTISNLEQEDIATYFCQQGNTLVHVRRGDQAGNK
```

IGKV03-05/IGKJ4

```
1        10        20        30        40        50        60        70        80        90        100       110
|        |         |         |         |         |   CDR
<-----------FR1----------><--CDR1--><------FR2------><2><---------------FR3----------------><--CDR3-><---FR4-->

DIVLTQSPASLAVSLGQRATISCRASESVDSYGNSFMHWYQQKPGQPPKLLIYRASNLESGIPARFSGSGSRTDFTLTINPVEADDVATYYCQQSNEDPFTFGSGTKLEIK
```

Figure S2: Flow cytometric gating of GP-binding B cells and light chain sequences.

A: Gating strategy for the identification of GPC$^+$GP1$^+$, GPC$^+$GP1$^-$ and GPC$^-$GP1$^-$B cells for adoptive transfer in the experiment to Fig. 2A-C.

B: Amino acid sequence of KL25 and of endogenous light chains cloned from the GPC$^+$GP1$^+$ B cell population of KL25H mice. Light chain (LC) sequences are outlined as amino acid translations with variable and joining regions identified by IgBLAST. Complementarity-determining regions (CDRs) according to IMGT nomenclature are marked in bold. Hypermutations in the KL25 light chain are displayed in red.

Figure S3

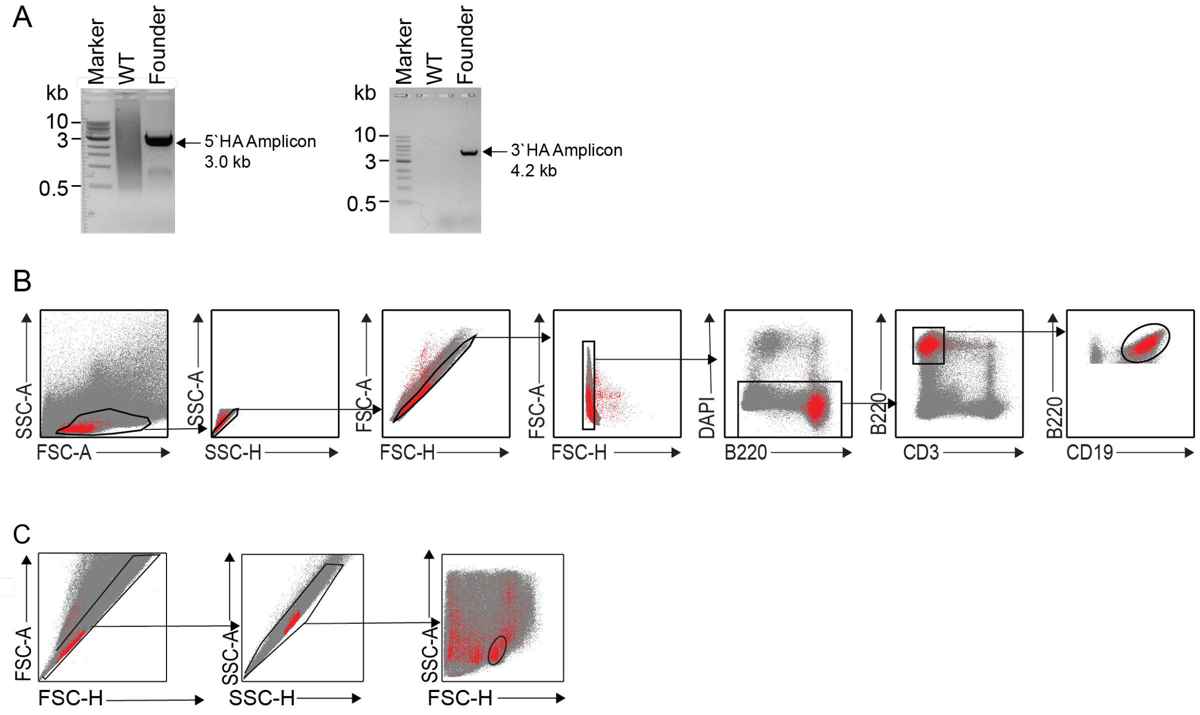

Figure S3: Identification of light chain knock-in founder mice by PCR and flow cytometric gating of splenic B cells as well as of bone marrow precursors.

A: Gel electrophoresis pictures of long-range PCR amplicons resulting from the genotyping of KL25 light chain knock-in founder mice. A PCR spanning from the inserted VJ into the genomic area outside of the 5' homology arm (left image) and a PCR spanning from the inserted VJ into the genomic area outside the 3' homology arm of the targeting construct (right image) were performed as schematically outlined in Fig. 3A.

B: Gating strategy for the identification of splenic B cells in Figs. 3C,E, 4A-D, 4K-L.

C: Gating for the subsequent identification of B cell progenitors in the bone marrow of mice.

Figure S4

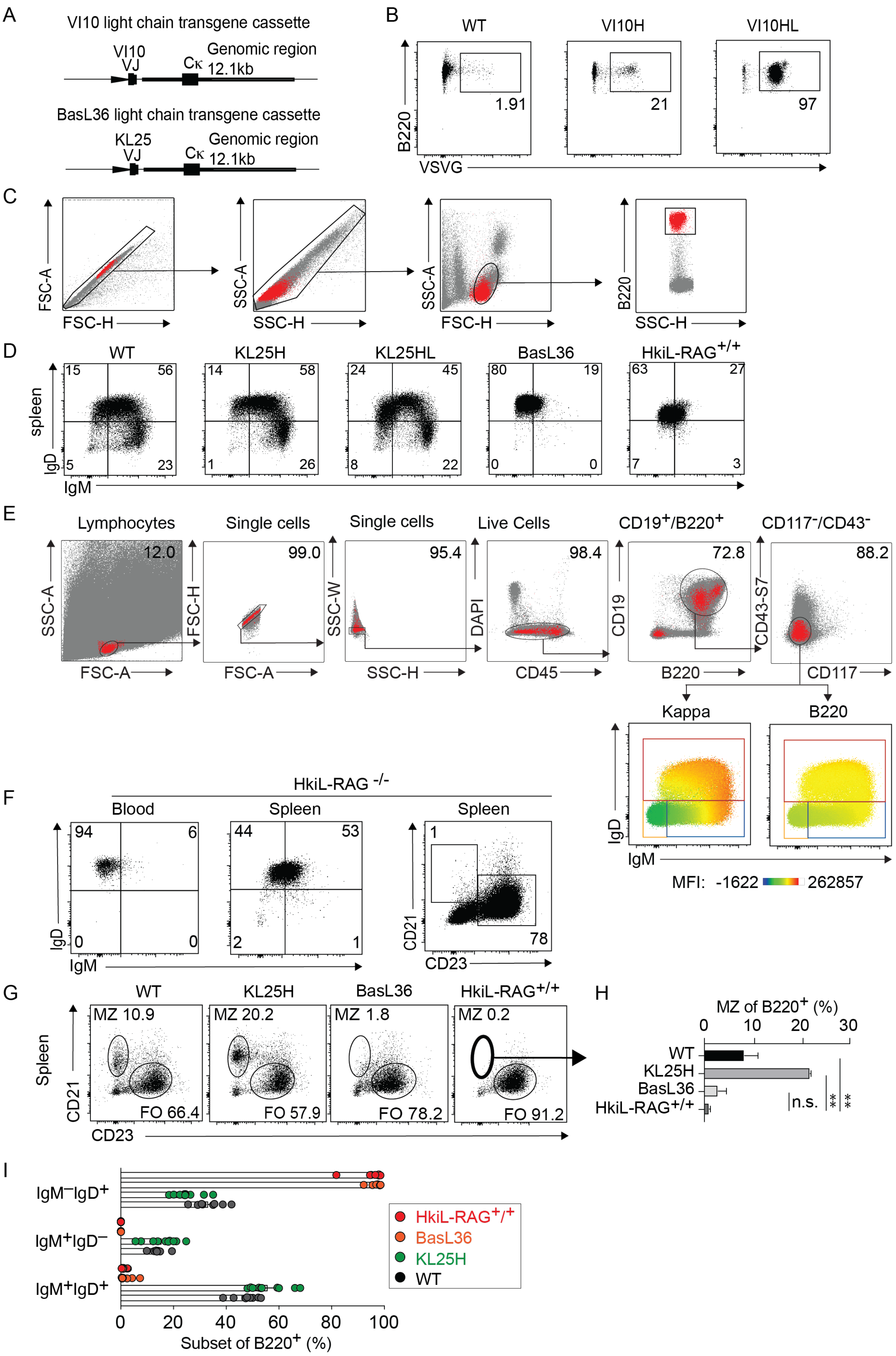
A
Vl10 light chain transgene cassette
Vl10 VJ
Cκ
Genomic region 12.1kb
BasL36 light chain transgene cassette
KL25 VJ
Cκ
Genomic region 12.1kb
B
WT
Vl10H
Vl10HL
1.91
21
97
B220
VSVG
C
FSC-A
FSC-H
SSC-A
SSC-H
SSC-A
FSC-H
B220
SSC-H
D
WT
KL25H
KL25HL
BasL36
HkiL-RAG+/+
spleen
IgD
IgM
E
Lymphocytes
Single cells
Single cells
Live Cells
CD19+/B220+
CD117-/CD43-
12.0
99.0
95.4
98.4
72.8
88.2
SSC-A
FSC-A
FSC-H
FSC-A
SSC-W
SSC-H
DAPI
CD45
CD19
B220
CD43-S7
CD117
Kappa
B220
IgD
IgM
MFI: -1622 262857
F
HkiL-RAG -/-
Blood
Spleen
Spleen
IgD
IgM
CD21
CD23
G
WT
KL25H
BasL36
HkiL-RAG+/+
MZ 10.9
MZ 20.2
MZ 1.8
MZ 0.2
FO 66.4
FO 57.9
FO 78.2
FO 91.2
Spleen
CD21
CD23
H
MZ of B220+ (%)
WT
KL25H
BasL36
HkiL-RAG+/+
n.s.
**
**
I
IgM−IgD+
IgM+IgD−
IgM+IgD+
HkiL-RAG+/+
BasL36
KL25H
WT
Subset of B220+ (%)

Figure S4: Light chain transgene cassettes and flow cytometric characterization of various BCR-engineered B cell populations.

A: Schematic of a transgene cassettes for expression of pre-rearranged VI10 and KL25 light chains in VI10HL and BasL36 mice, respectively. The targeting cassette consists of a 1000bp IgK8-28 promoter (triangle), leader peptide including intron, the rearranged VJ segments of the respective antibodies and 12.5kb downstream sequence, which comprises the C-kappa constant domain (Cκ).

B: Representative FACS plots showing VSV glycoprotein (VSVG) binding by peripheral blood B cells of WT, VI10 heavy chain knock-in (VI10H) and VI10 heavy chain knock-in plus VI10 light chain transgenic (VI10HL) mice. Numbers in plots show the percentage of VSVG binding cells among $B220^+$ B cells.

C: Gating strategy to identify $B220^+$ splenocytes phenotyped in the various mouse models in the experiments performed in Figures 4G,M, S4D and 6A,B.

D: Flow cytometric analysis of surface IgM and IgD expression by splenic B cells of the indicated KL25 BCR-expressing mouse strains.

E: Gating strategy to Fig. 4H-J. Heat plots report the Ig-kappa and B220 staining intensity, respectively, which were used as a basis to gate $IgD^-IgM^-B220^+$, $IgD^-IgM^+B220^+$ and $IgD^+IgM^+B220^+$ cells. Plots show concatenated samples from WT control mice.

F: Flow cytometric analysis of surface IgM and IgD expression by HkiL-$RAG^{-/-}$ B cells in blood and spleen, and paucity of splenic $CD23^{low}CD21^{high}$ marginal zone (MZ) B cells.

G,H: Representative FACS plots of $CD23^{low}CD21^{high}$ marginal zone (MZ) B cell and $CD23^{high}CD21^{low}$ follicular B cell (FO) subsets (G) of the indicated mouse genotypes.

Numbers in FACS plots represent the percentage of gated cells. One representative FACS plot from at least four mice analyzed in two independently conducted experiments is shown. Bars in (H) indicate the mean±SD of 4-6 mice from two combined experiments. One-way ANOVA with Dunnett's post-test was used for multiple comparisons in (H). **: $p < 0.01$, n.s.: not statistically significant.

I: Quantification of B cell subsets as displayed and gated in Fig. 4M. Symbols represent individual mice from three independently conducted experiments, bars indicate the mean±SEM.

Figure S5

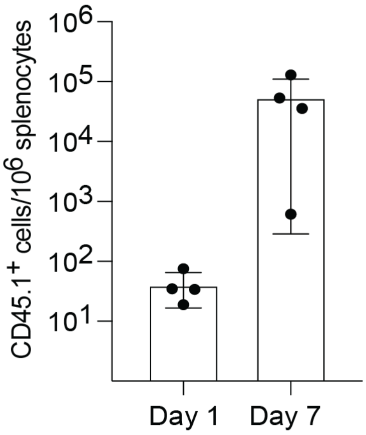

Figure S5: Early expansion of HkiL-RAG$^{-/-}$ B cells responding to rCl13/WE-N119S

Enumeration of adoptively transferred CD45.1^{+} HkiL-RAG$^{-/-}$ B cells in the experiment reported in Fig. 5H,I. Each symbol represents one animal. Bars indicate the mean±SD. Pooled data from two independently conducted experiments are shown.

Figure S6

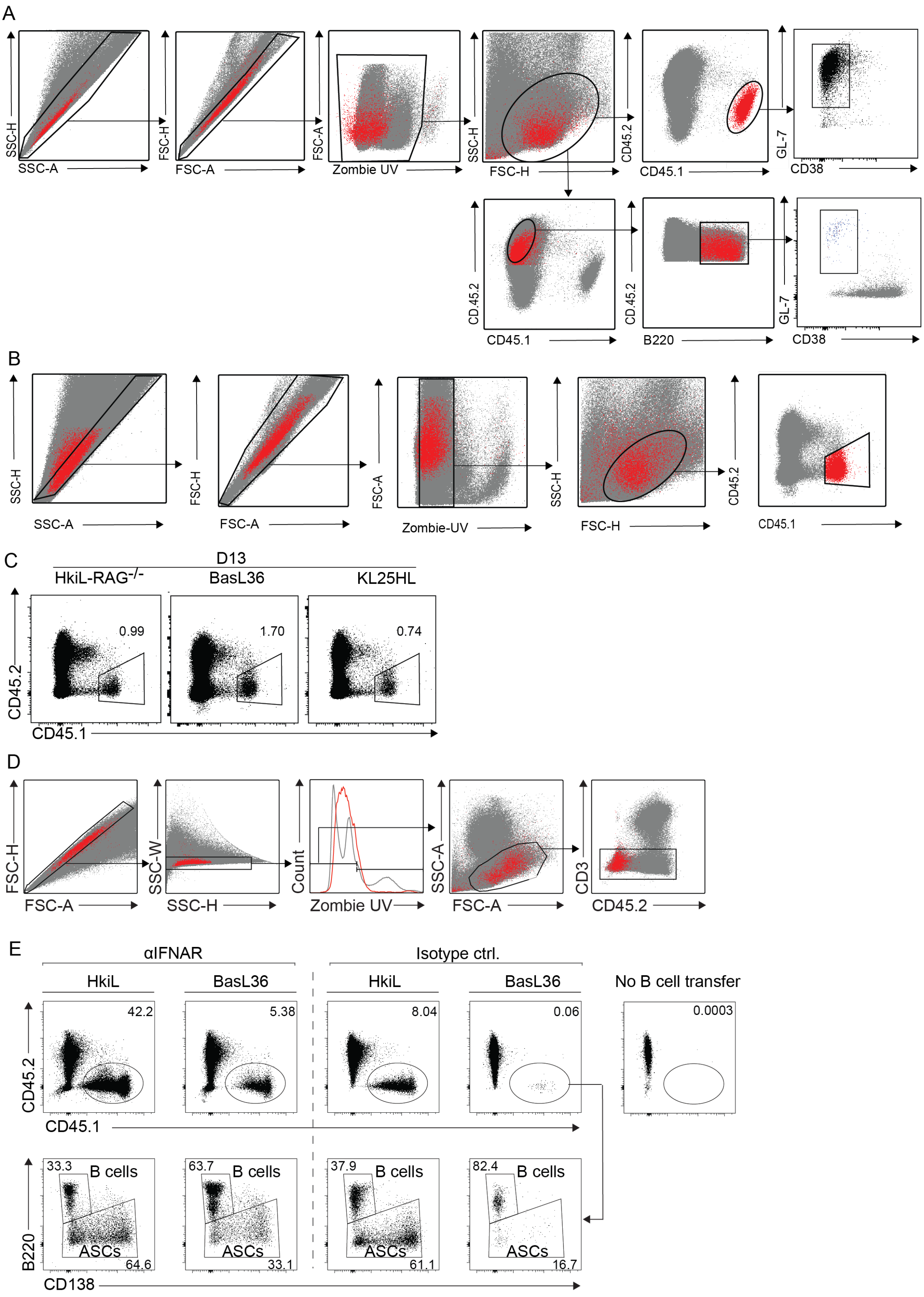
A
SSC-H
SSC-A
FSC-H
FSC-A
FSC-A
Zombie UV
SSC-H
FSC-H
CD45.2
CD45.1
GL-7
CD38
CD45.2
CD45.1
CD45.2
B220
GL-7
CD38
B
SSC-H
SSC-A
FSC-H
FSC-A
FSC-A
Zombie-UV
SSC-H
FSC-H
CD45.2
CD45.1
C
D13
HkiL-RAG-/-
BasL36
KL25HL
0.99
1.70
0.74
CD45.2
CD45.1
D
FSC-H
FSC-A
SSC-W
SSC-H
Count
Zombie UV
SSC-A
FSC-A
CD3
CD45.2
E
αIFNAR
Isotype ctrl.
HkiL
BasL36
HkiL
BasL36
No B cell transfer
42.2
5.38
8.04
0.06
0.0003
CD45.2
CD45.1
33.3
B cells
63.7
B cells
37.9
B cells
82.4
B cells
ASCs
64.6
ASCs
33.1
ASCs
61.1
ASCs
16.7
B220
CD138

Figure S6: Flow cytometric gating strategy, early expansion of HkiL, BasL36 and KL25HL B cells upon LCMV infection and their differential susceptibility to IFN-I-driven decimation at the onset of chronic infection.

A: Gating strategy for the identification of splenic progeny of adoptively transferred $CD45.1^+$ B cells in the experiments performed in Fig. 6E-G.

B: Gating strategy for the identification of adoptively transferred $CD45.1^+$ B cells and recipient $CD45.2^+$ B cells in spleen for the analyses in Fig. S6C.

C: Day 13 analysis of the experiment reported in Fig. 6C-G. Recipients ($CD45.2^+$) were infected with rCl13/WE and 6 days later received splenic B cells from HkiL-$RAG^{-/-}$, BasL36 or KL25HL mice. On day 13 two animals per group were sacrificed to analyze the expansion of the transferred cells in spleen following the gating strategy outlined in panel S6B. One representative FACS plot is shown. N = 2.

D: Flow cytometric analysis underlying the results reported in Fig. 6N-O. Pre-gating strategy for the analyses shown in (E). Red events in (D) correspond to the total pre-gated $CD45.1^+CD45.2^-$ population as identified in the top row of panel (E). One representative FACs plot is shown.

E: Concatenated FACS plots from n=4-5 mice underlying the data reported in Figure 6O. The top row displays the gating performed to assess the expansion of transferred B cells, the bottom row is pre-gated as shown in the upper row with gates set to assess the number of adoptively transferred ASC and B cell progeny. One control animal without adoptive B cell transfer (“no B cell transfer”) is shown for reference.

Numbers in FACS plots indicate the percentage of gated cells. N=2.
